# Comprehensive evaluation of the prevalent insulin resistance indices for pan-cancer incidence and mortality prediction

**DOI:** 10.1186/s41182-025-00884-5

**Published:** 2025-12-19

**Authors:** Shuhang Luo, Shengjie Lin, Li Ma, Runhua Tang, Ying Song, Li Ma, Jianye Wang

**Affiliations:** 1 Department of Urology, Beijing Hospital, National Center of Gerontology, Chinese Academy of Medical Sciences & Peking Union Medical College, Beijing, People’s Republic of China; 2 School of Medicine, Xiamen University, Xiamen, People’s Republic of China; 3 Department of Intensive Care Medicine, The Second Hospital & the Second Clinical Medical College of Lanzhou University, Lanzhou, People’s Republic of China; 4 Department of Radiation Oncology, The Third Affliated Hospital of Kunming Medical University (Yunnan Cancer Hospital, Yunnan Cancer Center), Kunming, People’s Republic of China

**Keywords:** Insulin resistance, Triglyceride‒glucose index, Pan-cancer incidence, Pan-cancer-specific mortality, Prospective study

## Abstract

**Background:**

Insulin resistance (IR) is increasingly recognized as a significant factor for cancer development and progression. While the triglyceride-glucose (TyG) index and its derivatives (TyG-BMI (body mass index), TyG-WC (waist circumference), and TyG-WHtR (waist-to-height ratio)) have been developed as reliable and straightforward surrogate tools for reflecting IR status, their comparative associations with pan-cancer incidence and mortality remain unclear. This study aimed to systematically evaluate the associations of these four IR-related indices with pan-cancer incidence and cancer-specific mortality in a large prospective cohort.

**Methods:**

This prospective cohort study analyzed data from 333,297 participants in the UK Biobank. The four IR-related indices mentioned above were calculated from baseline measurements. The primary outcomes were pan-cancer incidence and pan-cancer-specific mortality. Cox regression models, adjusted for demographic, socioeconomic, lifestyle, and clinical factors, were used to estimate hazard ratios (HRs) across participants' quartiles for each index. Besides, we assessed Dose–response relationships via restricted cubic splines (RCSs), and robustness via sensitivity and subgroup analyses.

**Results:**

Over a median follow-up of 15.2 years, 49,695 cases of different types of cancer and 12,852 cancer-specific deaths were recorded. All four IR-related indices showed significant non-linear associations with both outcomes (*p* < 0.001). After full adjustment, TyG-WC demonstrated the strongest and most graded association with pan-cancer incidence, with HRs progressively increasing from Q2 to Q4 (all *p* < 0.05 vs. Q1), and HR of Q4 was 1.11 (95% CI (confidence interval): 1.08, 1.15, *p* = 0.001). For pan-cancer-specific mortality, TyG-WC (HR = 1.37, 95% CI 1.28, 1.46; *p* < 0.001), TyG-WHtR (HR = 1.25, 95% CI 1.18, 1.33; *p* < 0.001), and TyG-BMI (HR = 1.22, 95% CI 1.15,1.29; *p* < 0.001) were significantly elevated in Q4, with TyG-WC again showing a significant dose–response trend across all quartiles. In contrast, the original TyG index showed the weakest predictive performance. Subgroup analyses indicated effect modifications by sex, smoking status, and comorbidities. Sensitivity analyses confirmed the robustness of the associations, particularly for TyG-WC.

**Conclusion:**

IR-related indices, especially TyG-WC, are significantly associated with both pan-cancer incidence and cancer-specific mortality. Compared with TyG, TyG-BMI, and TyG-WHtR, TyG-WC demonstrated stronger associations, suggesting its potential utility for stratifying cancer risk and prognosis in clinical and public health settings.

**Supplementary Information:**

The online version contains supplementary material available at 10.1186/s41182-025-00884-5.

## Introduction

Cancer remains a leading global cause of mortality, responsible for approximately 10 million annual deaths worldwide and over 600,000 deaths in the United States in 2025 [1, 2], with its burden projected to rise significantly due to population aging [3, 4]. Growing evidence indicates that metabolic dysfunction, particularly hyperinsulinemia, is strongly associated with increased cancer incidence and mortality rates [5–8]. Insulin resistance (IR), a hallmark feature of diabetes and obesity, is characterized by diminished responsiveness of insulin-sensitive tissues to insulin stimulation [9]. Current research has established that IR is a critical factor in the pathogenesis of various disorders, including cardiovascular disease (CVD), metabolic syndrome, non-alcoholic fatty liver disease (NAFLD), atherosclerosis, and notably, cancer development and progression [10–12].

In addition to established but costly or limited-availability methods, such as the hyperinsulin-euglycemic clamp and the Homeostasis Model Assessment of Insulin Resistance (HOMA-IR) [13–15], the Triglyceride-glucose (TyG) index has emerged as a simple yet reliable surrogate marker for IR [16, 17]. The TyG index, which is calculated from fasting blood triglyceride (TG) and glucose, demonstrates comparable diagnostic accuracy for IR while offering superior clinical practicality [18, 19]. Increasing evidence indicates strong connections between the TyG index and various kinds of malignancies, including bladder, prostate, breast, lung, gastric, and colorectal cancers, highlighting its possible involvement in their progression [20–27]. While the effect size of IR may vary across cancer types, a pan-cancer analysis is biologically justified given the pleiotropic nature of its underlying mechanisms, including hyperactivation of the insulin/IGF-1(Insulin-like Growth Factor 1) axis, inflammation, oxidative stress, and adipokine dysregulation [28–32]. Consistent with this rationale, large-scale meta-analyses demonstrate that higher TyG index levels are significantly associated with increased pan-cancer risk [33, 34]. Recent methodological improvements have further refined the TyG index by incorporating anthropometric parameters, yielding derivative indices such as TyG-BMI (BMI-adjusted), TyG-WC (waist circumference-adjusted), and TyG-WHtR (waist-to-height ratio-adjusted). While all of these indices are derived from the foundational TyG index, these modified indices exhibit differential predictive capacities for various diseases. For example, TyG-WC may better reflect NAFLD, whereas TyG-WHtR appears to be more sensitive for sarcopenic obesity [35, 36]. However, to date, only one study has systematically evaluated these four indices (TyG, TyG-BMI, TyG-WC, and TyG-WHtR) specifically regarding esophageal cancer [37]. A critical knowledge gap persists regarding the simultaneous comparison of TyG, TyG-BMI, TyG-WC, and TyG-WHtR for assessing their association with both pan-cancer incidence and mortality, leaving the optimal indicator unclear.

This prospective cohort study, which utilized data from the UK Biobank, was designed to systematically examine the potential associations between four IR-related indices (TyG, TyG-BMI, TyG-WC, and TyG-WHtR) and both pan-cancer incidence and cancer-specific mortality. The analysis incorporated extensive adjustment for potential confounding variables and included comprehensive sensitivity analyses to assess the robustness of the observed relationships.

## Methods

### Study design and participants

This prospective, population-based study analyzed data from the UK Biobank [38], a large-scale cohort comprising over 500,000 participants aged 37–73 years recruited between 2006 and 2010 across England, Scotland, and Wales. At baseline, participants completed touchscreen questionnaires, underwent standardized physiological measurements, and provided biological samples. Longitudinal follow-up included linkage to national hospital admissions, cancer registries, and mortality records. Participants who died from non-cancer causes or were lost to follow-up were censored at the date of death or loss to follow-up. The design and conduct of this study adhered to the STROBE (Strengthening the Reporting of Observational Studies in Epidemiology) reporting guidelines [39]. The completed STROBE checklist is provided as a supplementary file.

**Fig. 1:**
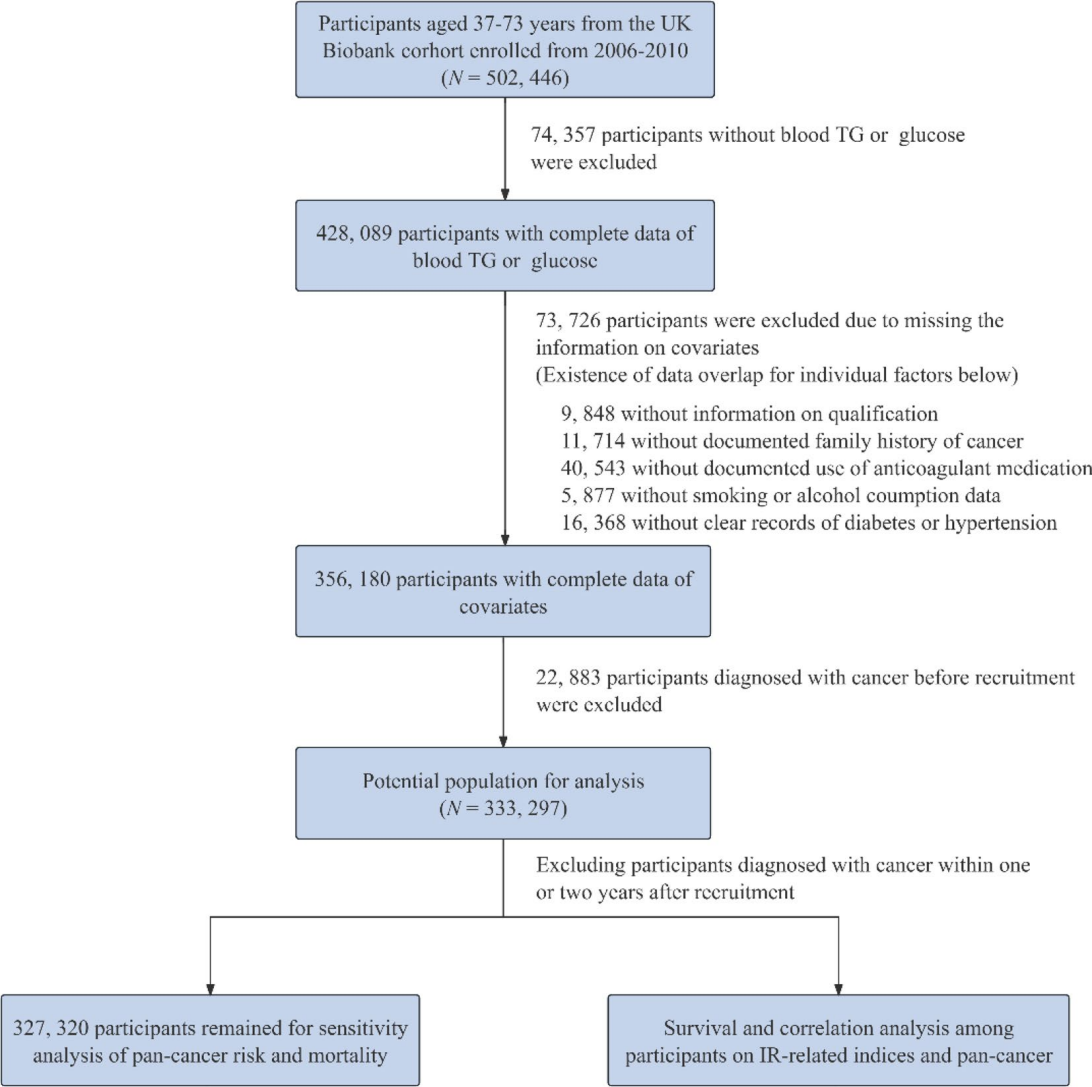
Flowchart of selecting the study population in the UK Biobank. *TG* triglyceride, *IR* insulin resistance

To evaluate the predictive performance of the TyG index, TyG-BMI, TyG-WC, and TyG-WHtR for cancer incidence and cancer-specific mortality, we analyzed data from 502,446 participants in the UK Biobank study. To minimize potential bias from missing data, we performed a complete-case analysis. We excluded individuals with missing values for TG or glucose (*n* = 74,357). We further excluded participants with incomplete data on any of the covariates (*n* = 73,726), which included 9848 individuals with missing information on educational qualification, 11,714 with unknown family history of cancer, 40,543 with missing data on anticoagulant medication use, 5877 with incomplete smoking or alcohol history, and 16,368 lacking a clear record of previous diagnosis of diabetes or hypertension. It should be noted that these categories are not mutually exclusive, as some individuals had more than one type of missing data. Additional exclusions were applied to participants with a baseline cancer diagnosis (*n* = 22,883), resulting in a final analytical cohort of 333,297 participants. Details on participant selection are illustrated in Fig. 1.

### Exposure

Following a standardized fasting period, fasting plasma glucose, TG, and high-density lipoprotein cholesterol (HDL-C) were measured on a Beckman Coulter AU5800 automated analyzer using standardized protocols. All blood samples were processed immediately after collection, with the plasma/serum components separated and stored at − 80 °C until analysis. Detailed analytical procedures and quality control measures are available through the UK Biobank resource (Reference ID: 1227). Body measurement indicators, such as height and weight, were measured by professionals for the participants.

We evaluated four IR-related indices derived from measurements as follows according to established methodologies [40–42]:

- TyG index: Ln [fasting TG (mg/dL) × fasting glucose (mg/dL)/2]
- TyG-BMI index: TyG index × body mass index (kg/m^2^) [weight (kg)/height^2^ (m.^2^)]
- TyG-WC index: TyG index × waist circumference (cm)
- TyG‒WHtR index = TyG index × [waist circumference (cm)/height (cm)]

The conversion of TG from mmol/L to mg/dL is done using a conversion formula: 1 mmol/L equals 88.5 mg/dL, and blood glucose is converted from mmol/L to mg/dL using a conversion formula: 1 mmol/L = 18 mg/dL.

### Assessment of outcomes

Cancer diagnoses were ascertained via ICD-10 codes, encompassing malignancies across multiple systems: digestive (anal [C21], colorectal [C18-C20], esophageal [C15], gastric [C16], liver [C22], pancreatic [C25], small intestinal [C17]), genitourinary (bladder [C67], kidney [C64],, uterine [C54-C55], ovarian [C56], prostate [C61]), thoracic (lung [C33-C34], mesothelioma [C45]), hematologic (leukemia [C91-C95], lymphoma [C81-C86,C88], multiple myeloma [C90]), and other major sites (brain [C71], breast [C50], laryngeal [C32], melanoma [C43-C44], oral [C00-C14], soft tissue [C46-C49], and thyroid [C73]).

Cancer-specific mortality was determined using the UK Biobank Death Register, which provides verified mortality data, including the exact date of death (Field ID 40000) and the underlying cause of death coded according to ICD-10 (Field ID 40001). These records were linked with official national death registries: NHS Digital for England and Wales (data complete through May 30, 2024) and the National Records of Scotland (data complete through December 31, 2023).

### Covariates

This study examined a range of demographic characteristics at baseline, including age (analyzed as a continuous variable), genetic sex (male/female), ethnicity (White/non-White), and educational attainment (College/University degree or not). Socioeconomic status was assessed by the Townsend Deprivation Index (continuous, derived from postal code data on unemployment, car and home ownership, and household overcrowding, with higher scores indicating greater deprivation), which was subsequently divided into quartiles (Q1-Q4) for analysis. Health behaviors included smoking status (classified as never/ever smoked) and alcohol consumption (never/ever drank). Clinical factors comprised the family history of cancer (yes/no, determined through baseline records of cancer diagnoses in participants' parents and siblings), anticoagulant use (yes/no), and histories of heart failure, hypertension, CVD, and stroke (ascertained through ICD-10 codes in the UK Biobank's linked inpatient hospital records using established diagnostic criteria [43]). All variables were dichotomized as present or absent based on baseline self-reports, except when objective measures (e.g., ICD codes) were available.

### Statistical analysis

The baseline characteristics were reported as medians with interquartile ranges (IQR) for continuous variables and percentages (%) for categorical variables. The Shapiro–Wilk test, P-P plots, and Q-Q plots were used to assess the normality of continuous variables. Differences between groups were analyzed using t-tests for normally distributed continuous variables, Kruskal–Wallis rank-sum tests for non-normally distributed continuous variables, and chi-square tests for categorical variables. Cox proportional hazards regression models were applied to examine the relationships between TyG, TyG-BMI, TyG-WC, and TyG-WHtR scores (with the ideal state as the reference group) and outcomes, including pan-cancer incidence and pan-cancer-specific mortality. The proportional hazards assumption was checked using Schoenfeld residuals; the global test indicated no substantial violations for the primary exposure variables (all *p* > 0.05). For covariates with minor violations, transient covariates were incorporated into the model as a sensitivity analysis, without altering the primary findings. Multicollinearity among the covariates was evaluated using the variance inflation factor; values below 10 were deemed acceptable. The final model, adjusted for age, sex, ethnicity, education, socioeconomic status, smoking status, alcohol consumption, family history of cancer, history of anticoagulant use, and history of hypertension, CVD, stroke, heart failure, and diabetes, calculated the hazard ratios (HRs) and 95% confidence intervals (CIs) for each IR score about the outcome variables. The model sequence was as follows: Model 1: Unadjusted. Model 2: Adjusted for fundamental sociodemographic factors: age, sex, ethnicity, educational level, and Townsend deprivation index. Model 3: Further adjusted for lifestyle factors (alcohol consumption, smoking status) and key metabolic comorbidities (history of diabetes, hypertension), and recruitment center. Model 4: Further adjusted for anticoagulant history and family history of cancer (a proxy for genetic susceptibility). Model 5: Fully adjusted model, additionally incorporating history of CVD, stroke, and heart failure. The models were revised to incorporate sociodemographic factors, lifestyle habits, metabolic disorders, genetic factors, and medication usage. Subsequently, modifications were applied for cardiovascular comorbidities to distinguish confounding variables from mediation and to ascertain the independent impact of IR.

In addition, the cumulative hazard of pan-cancer incidence and pan-cancer-specific mortality across quartiles of IR-related indices was compared using the Kaplan–Meier method and statistically assessed using the log-rank test. Additionally, restricted cubic spline (RCS) models were applied to examine the dose–response relationship between four IR-related indices and pan-cancer incidence and pan-cancer-specific mortality. For the RCS analysis, the number of knots was set at three, with their positions set at the default values of the 10th, 50th, and 90th percentiles. The nonlinearity of the associations was formally tested using the likelihood ratio test, comparing the model with RCS terms against one that included the index as a linear term. A significant p-value (< 0.05) from this test was interpreted as evidence of a non-linear dose–response relationship.

We conducted extensive subgroup analyses to evaluate the robustness of our findings and examine potential effect modifiers. Participants were stratified by key demographic and clinical characteristics, including age (< 60 years vs. ≥ 60 years), sex (male vs. female), BMI (< 25 kg/m^2^ vs. ≥ 25 kg/m^2^), smoking status (yes vs. no), hypertension (yes vs. no), diabetes (yes vs. no), family history of cancer (yes vs. no), and histories of heart failure (yes vs. no), stroke (yes vs. no), and CVD (yes vs. no). Statistical differences between subgroups were assessed using interaction terms.

To ensure a detailed assessment of our results, we performed extensive sensitivity analyses to address possible confounding factors. During these analyses, we omitted participants whose cancer diagnoses or fatalities took place within one or two years of enrollment. For all analyses involving multiple comparisons, we used the Bonferroni method to adjust P values. All reported P values have been corrected accordingly. All statistical analyses were performed using R version 4.3.2 (R Foundation for Statistical Computing), with two-sided tests and a significance threshold of *p* < 0.05.

## Results

### Participant characteristics

Table 1 presents the baseline characteristics of the 333,297 participants. The cohort had a median age of 56.00 years (IQR (49.00, 62.00)), with a slight female predominance (53.7%). Ethnically, the majority of participants were White (94.4%), followed by Asian (2.1%) and African (0.7%) participants. Regarding educational attainment, 39.6% of participants held a college or university degree. Compared with cancer-free individuals, those with a cancer diagnosis were more likely to be older, male, White, and from England; to have lower education and income; to smoke and consume alcohol more frequently; and to have a history of hypertension, diabetes, CVD, or a family history of cancer (all *P* < 0.001). In addition, these patients had higher baseline TG levels, TG/HDL-C ratios, and IR indices (all *P* < 0.001). Similar findings were also observed among individuals who died from pan-cancer–specific causes compared with those who did not.

**Table 1:** Baseline characteristics of the study population

Variables	Total population	Pan-cancer incidence	No	P-value	Pan-cancer-specific mortality	No	P-value
		Yes			Yes		
Age, years	56.00 (49.00,62.00)	61.00(55.00,65.00)	55.00 (48.00,61.00)	< 0.001	62.00(57.00,66.00)	56.00(49.00,62.00)	< 0.001
Sex				< 0.001			< 0.001
Female	179,063(53.7)	23,662(47.6)	155,401(54.8)		5848(45.5)	173215(54.1)	
Male	154,234(46.3)	26,033(52.4)	128,201(45.2)		7004(54.5)	147230(45.9)	
Ethnicity				< 0.001			< 0.001
White	314,508(94.4)	48,064(96.7)	266,444(93.9)		12,387(96.4)	302,121(94.3)	
Non-white	18,789(5.6)	1,631(3.3)	17,158(6.1)		465(3.6)	18,324(5.7)	
Center				< 0.001			< 0.001
England	295225(88.6)	44440(89.4)	250785(88.4)		11162(86.8)	284063(88.7)	
Scotland	23627(7.1)	3080(6.2)	20547(7.3)		1091(8.5)	22536(7.0)	
Wales	14445(4.3)	2175(4.4)	12270(4.3)		599(4.7)	13846(4.3)	
Educational levels				< 0.001			< 0.001
College or University degree	132,016(39.6)	18862(38.0)	113154(39.9)		4571(35.6)	127445(39.8)	
Less than a college degree	201,281(60.4)	30833(62.0)	170448(60.1)		8281(64.4)	193000(60.2)	
Townsend deprivation index	– 2.33(– 3.75, 0.14)	– 2.50(– 3.83, – 0.21)	– 2.30(– 3.73, 0.19)	< 0.001	– 2.33(– 3.75, 0.13)	– 2.31(– 3.71, 0.24)	0.048
Smoking status				< 0.001			< 0.001
Yes	32226(9.7)	5164(10.4)	27062(9.5)		2056(16.0)	30170(9.4)	
No	301071(90.3)	44531(89.6)	256540(90.5)		10796(84.0)	290275(90.6)	
Alcohol consumption				< 0.001			< 0.001
Yes	310482(93.2)	46511(93.6)	263971(93.1)		11871(92.4)	298611(93.2)	
No	22815(6.8)	3184(6.4)	19631(6.9)		981(7.6)	21834(6.8)	
Family cancer history				< 0.001			< 0.001
Yes	114086(34.2)	19135(38.5)	94951(33.5)		5044(39.2)	109042(34.0)	
No	219211(65.8)	30560(61.5)	188651(66.5)		7808(60.8)	211403(66.0)	
Hypertension history				< 0.001			< 0.001
Yes	173249(52.0)	29782(59.9)	143467(50.6)		8318(64.7)	164931(51.5)	
No	160048(48.0)	19913(40.1)	140135(49.4)		4534(35.3)	155514(48.5)	
Diabetes history				< 0.001			< 0.001
Yes	29023(8.7)	6131(12.3)	22892(8.1)		2076(16.2)	26947(8.4)	
No	304274(91.3)	43564(87.7)	260710(91.9)		10776(83.8)	293498(91.6)	
CVD history				< 0.001			< 0.001
Yes	44121(13.2)	9919(20.0)	34202(12.1)		3051(23.7)	41070(12.8)	
No	289176(86.8)	39776(80.0)	249400(87.9)		9801(76.3)	279375(87.2)	
TG, mmol/L	1.45(1.02,2.11)	1.52(1.07,2.17)	1.44(1.01,2.10)	< 0.001	1.58(1.12, 2.24)	1.44(1.02, 2.10)	< 0.001
HDL-C, mmol/L	1.41(1.18,1.69)	1.39(1.16,1.67)	1.41(1.18,1.69)	< 0.001	1.36(1.13,1.64)	1.41(1.18,1.69)	< 0.001
TG/HDL-C ratio	2.35(1.47,3.87)	2.51(1.56,4.04)	2.33(1.45,3.84)	< 0.001	2.70(1.67,4.29)	2.34(1.46,3.85)	< 0.001
TyG index	8.65(8.28,9.05)	8.71(8.34,9.09)	8.64(8.28,9.04)	< 0.001	8.76(8.39,9.14)	8.65(8.28,9.05)	< 0.001
TyG-BMI index	230.68(202.44, 264.32)	234.35(206.45,267.24)	230.02(201.76,263.78)	< 0.001	239.52(210.21,273.44)	230.33(202.14,263.91)	< 0.001
TyG-WC index	774.01(672.86,877.53)	795.93(696.44,895.28)	769.98(668.94,874.03)	< 0.001	814.80(712.49, 918.32)	772.38(671.25, 875.67)	< 0.001
TyG-WHtR index	4.56(4.03,5.13)	4.67(4.14,5.22)	4.55(4.01,5.12)	< 0.001	4.79(4.24,5.35)	4.56(4.02,5.13)	< 0.001

Data are presented as the median (IQR) for continuous variables and n (%) for categorical variables

*CVD* cardiovascular disease, TG triglyceride, *HDL-C* high-density lipoprotein cholesterol, *TG/HDL-C* triglyceride-to-high-density lipoprotein cholesterol, *TyG* triglyceride-glucose, *BMI* body mass index, *WC* waist circumference, *WHtR* weight-to-height ratio

### Associations between IR-related indices and pan-cancer incidence and pan-cancer-specific mortality

**Fig. 2:**
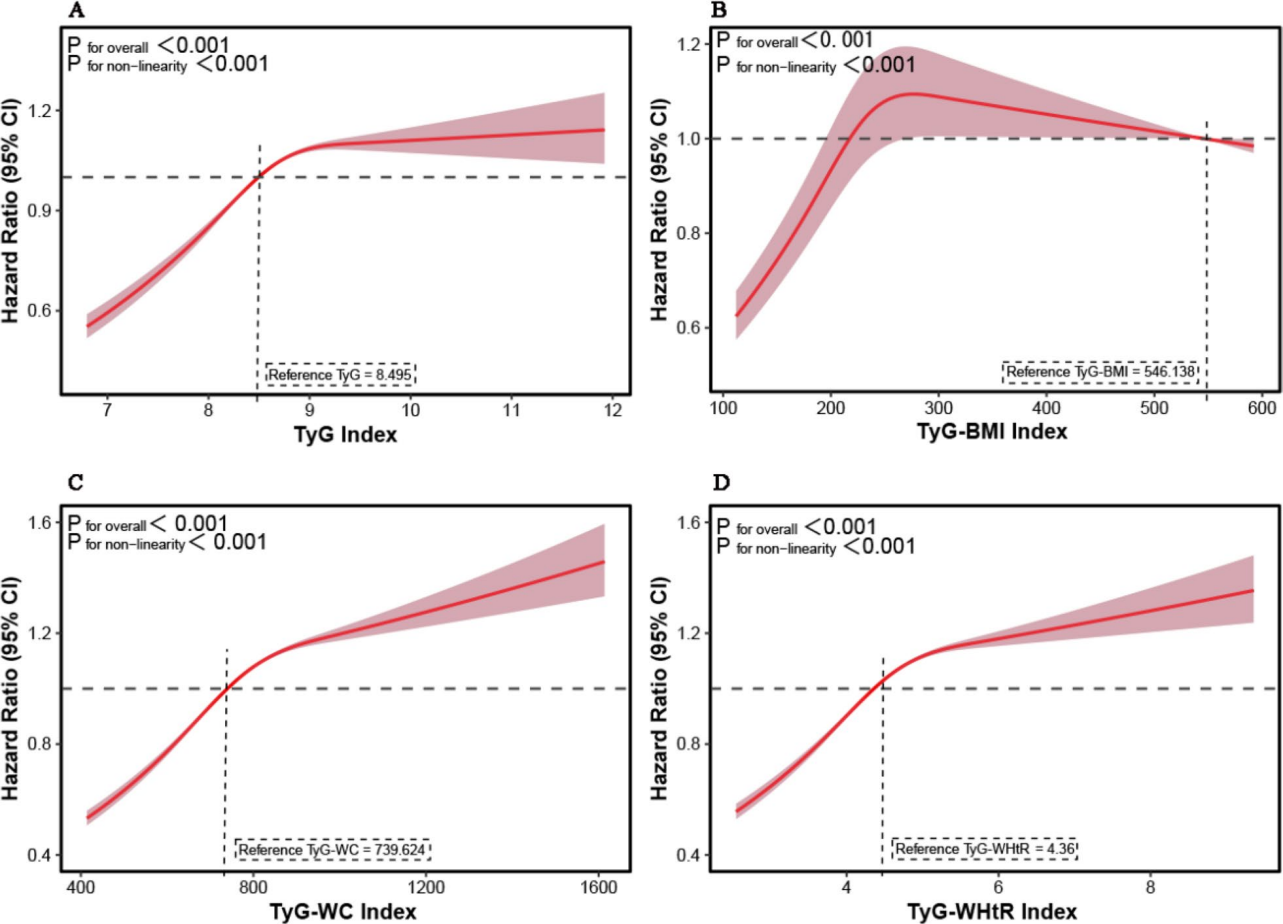
Dose–response relationship of IR-related indices with the risk of pan-cancer incidence. **A**: TyG index; **B**: TyG-BMI index; **C**: TyG-WC index; **D**: TyG-WHtR index. *IR*: insulin resistance, *TyG* triglyceride-glucose index, *BMI* body mass index, *WC* waist circumference, *WHtR* waist-to-height ratio

**Fig. 3:**
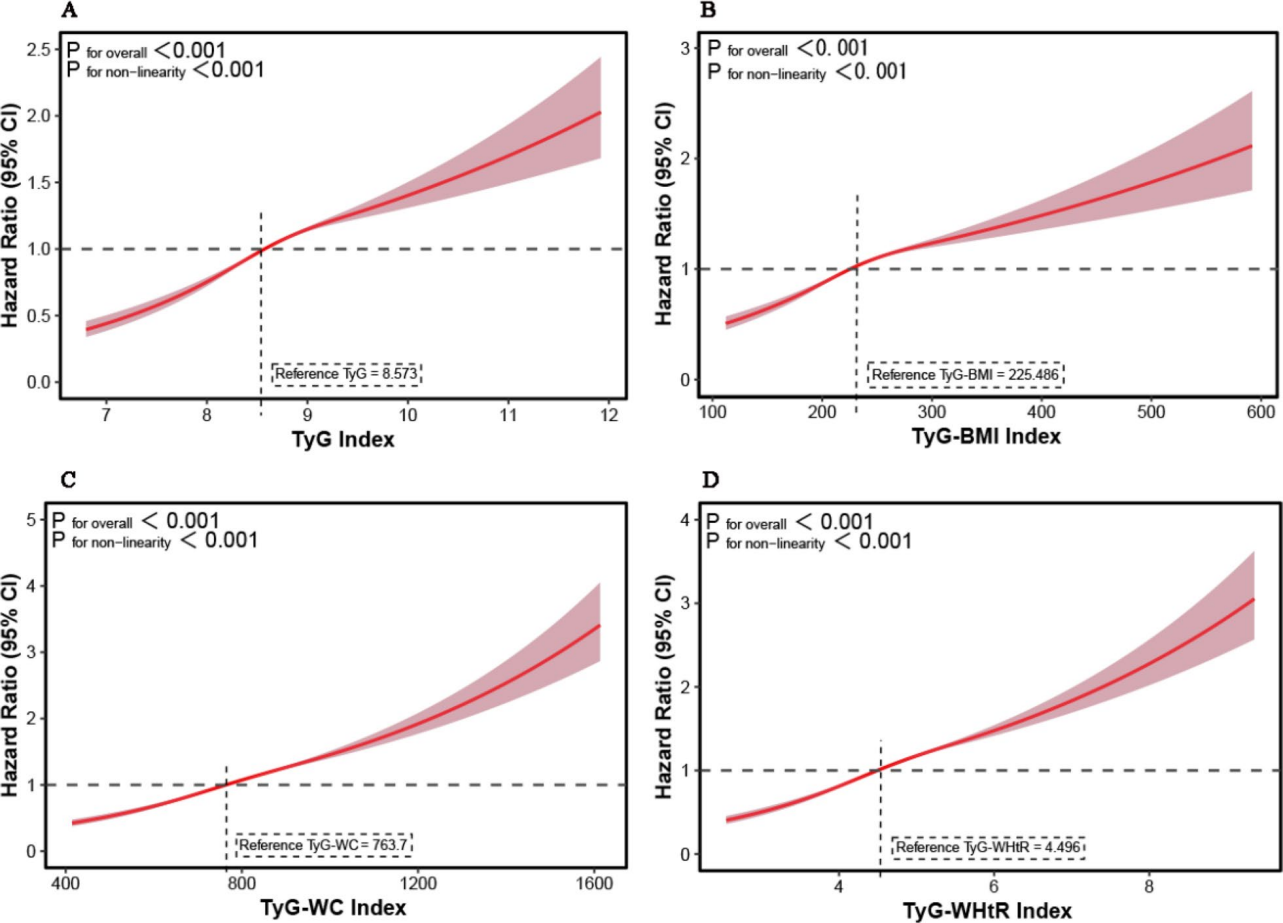
Dose–response relationship of IR-related indices with the risk of pan-cancer-specific mortality. **A**: TyG index; **B**: TyG-BMI index; **C**: TyG-WC index; **D**: TyG-WHtR index. *IR*: insulin resistance, *TyG* triglyceride-glucose index, *BMI* body mass index, *WC* waist circumference, *WHtR* waist-to-height ratio

The UK follow-up durations varied: England and Wales had data complete until May 30, 2024, while Scotland's data was finalized by December 31, 2023. Over a median follow-up of 15.2 years, there were 49,695 new pan-cancer cases (14.9% of the population) and 12,852 deaths directly linked to pan-cancer (3.9% of cases). Figure 2 and Fig. 3 illustrate the dose‒response relationships between IR-related indices and the risk of pan-cancer incidence and pan-cancer-specific mortality. RCS analyses revealed a non-linear relationship between the four IR-related indices (TyG index, TyG-BMI index, TyG-WC index and TyG-WHtR index) and pan-cancer incidence (all *P* < 0.001, Fig. 2). Additionally, there was a non-linear relationship between the four IR-related indices (TyG index, TyG-BMI index, TyG-WC index and TyG-WHtR index) and pan-cancer-specific mortality (all *P* < 0.001, Fig. 3). The RCS analyses revealed a progressive increase in pan-cancer–specific mortality risk with increasing IR index levels, a trend that was consistently observed across all four indices, with the association being particularly pronounced for the TyG-WC and TyG-WHtR indices (Fig. 3A–D). Similarly, as the TyG-WC index (Fig. 2C) and TyG-WHtR index (Fig. 2D) increased, the hazard ratios for pan-cancer incidence also gradually increased.

**Fig. 4:**
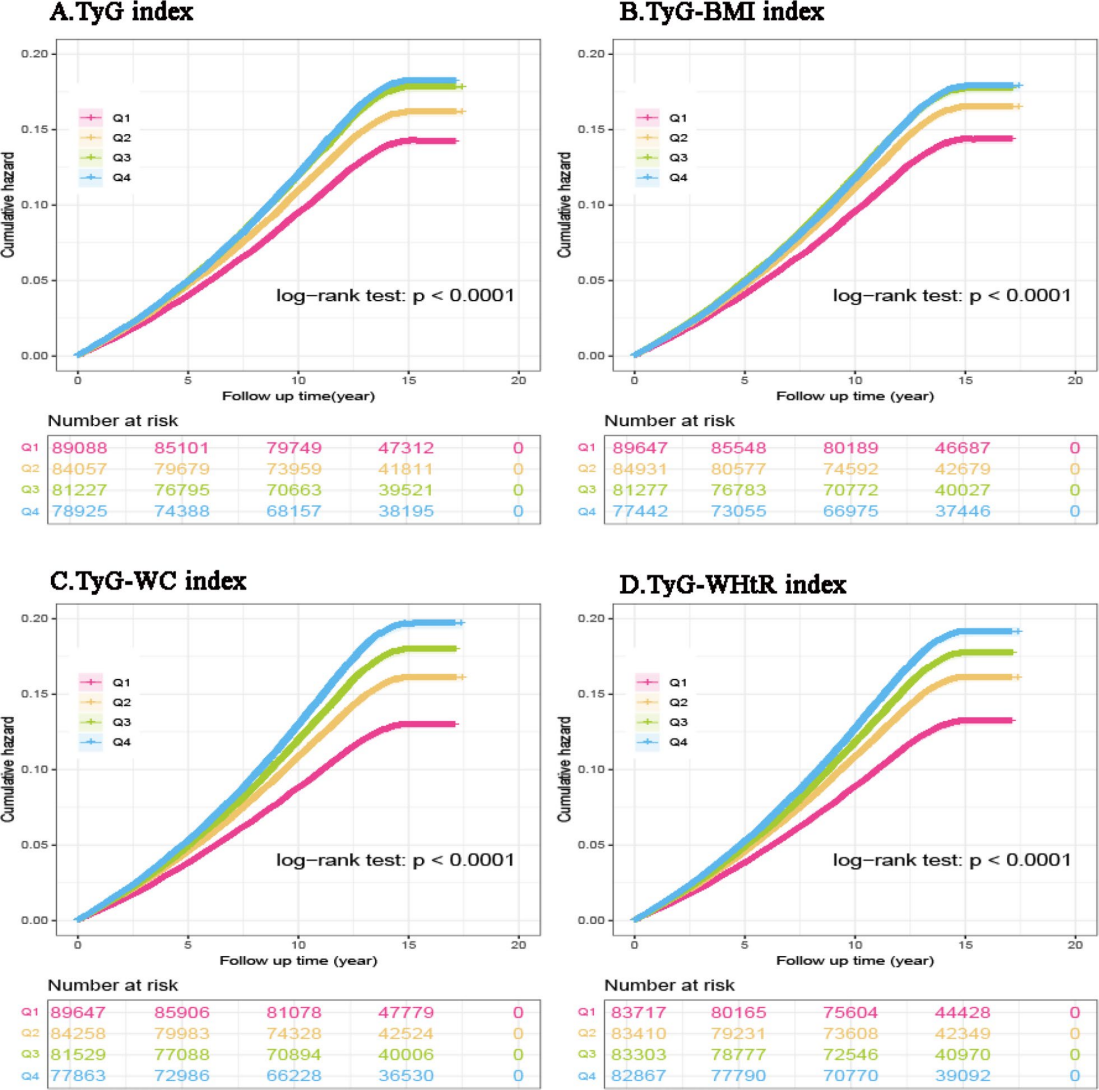
Kaplan–Meier curves of pan-cancer incidence stratified by the quartiles of IR-related indices. *IR*: insulin resistance, *TyG* triglyceride-glucose index, *BMI* body mass index, *WC* waist circumference, *WHtR* waist-to-height ratio

**Fig. 5:**
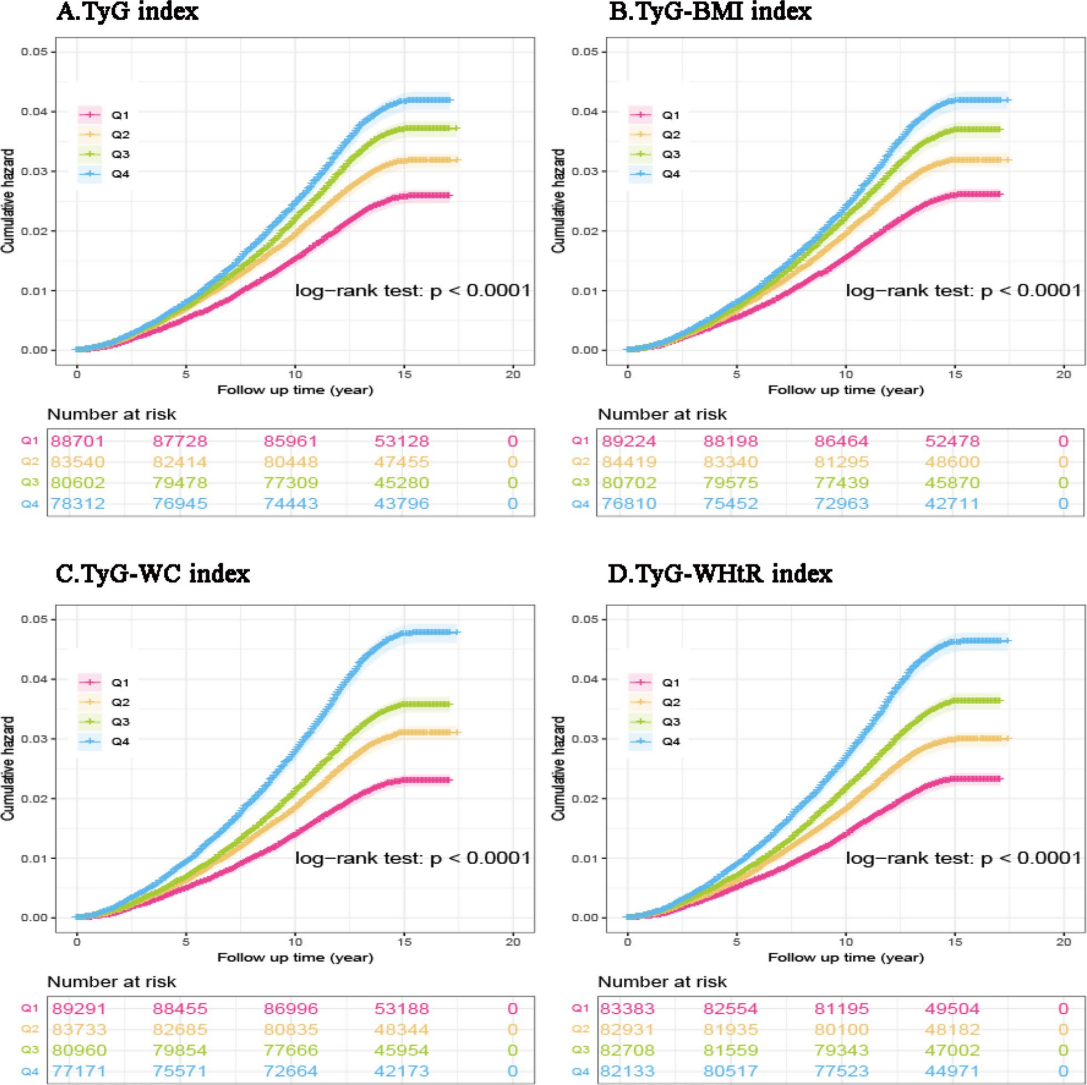
Kaplan–Meier curves of pan-cancer-specific mortality stratified by the quartiles of IR-related indices. 2 *IR*: insulin resistance, *TyG* triglyceride-glucose index, *BMI* body mass index, *WC* waist circumference, *WHtR* waist-to-height ratio

The IR-related indices were subsequently treated as continuous variables and divided into quartiles, with the first quartile (Q1) serving as a reference. Kaplan–Meier curves of pan-cancer incidence and pan-cancer-specific mortality stratified by the quartiles of IR-related indices (TyG, TyG-BMI, TyG-WC, TyG-WHtR) are presented in Fig. 4 and Fig. 5. Kaplan–Meier curves also revealed an increasing trend in pan-cancer incidence and pan-cancer-specific mortality with increasing IR-related indices. For all four IR-related indices, the cumulative hazards of pan-cancer incidence and pan-cancer-specific mortality were significantly greater among participants in the Q2-Q4 categories than among those in the Q1 categories (all log-rank tests *P* < 0.001; Figs. 4 and 5).

We employed multivariable Cox proportional hazards regression with five progressively adjusted models to examine the relationships between IR-related indices and pan-cancer incidence and pan-cancer–specific mortality. As Table 2 shows, regarding pan-cancer incidence, after full adjustment in Model 5, we found a significantly elevated risk in the highest quartile (Q4) for TyG -BMI (HR = 1.05, 95% CI 1.02, 1.07; *p* = 0.001), TyG-WC (HR = 1.11, 95% CI 1.08, 1.15; *p* < 0.001), and TyG-WHtR (HR = 1.05, 95% CI 1.02, 1.08; *p* < 0.001) compared with the reference group (Q1). Notably, for TyG-WC, all higher quartiles (Q2-Q4) presented a marked, stepwise increase in risk compared with that of the Q1 group (all *p* < 0.05, 1.11 (1.08, 1.15)). Interestingly, for TyG, the Q2 group presented a modest but significant reduction in pan-cancer incidence (HR = 0.96, 95% CI 0.94, 0.98; *p* = 0.002) relative to the reference group (Q1). In addition, Table 3 shows the associations between IR-related indices and the risk of pan-cancer-specific mortality. In fully adjusted Cox models (Model 5), we found significantly elevated risk in the highest quartile (Q4) for all four IR-related indices: TyG (HR = 1.10, 95% CI 1.04, 1.16; *p* = 0.001), TyG-BMI (HR = 1.22, 95% CI 1.15,1.29; *p* < 0.001), TyG-WC (HR = 1.37, 95% CI 1.28, 1.46; *p* < 0.001), and TyG-WHtR (HR = 1.25, 95% CI 1.18, 1.33; *p* < 0.001) compared with the reference group (Q1). Notably, as the TyG-WC score increased progressively, the risk of pan-cancer–specific mortality also increased accordingly (HR of Q2 = 1.07, HR of Q3 = 1.10, and HR of Q4 = 1.37; all *p* < 0.05). The C-indexes of TyG-related indices for pan-cancer incidence were: 0.649 (95% CI 0.647–0.652) for the TyG index, 0.650 (95% CI 0.647–0.652) for the TyG-BMI and TyG-WhtR index, and 0.650 (95% CI 0.648–0.652) for the TyG-WC index. Meanwhile, the C-index of TyG-related indices for pan-cancer-specific mortality was: 0.705 (95% CI 0.700–0.709) for the TyG index, 0.706 (95% CI 0.702–0.711) for the TyG-BMI index, 0.707 (95% CI 0.703–0.712) for the TyG-WC, and 0.707 (95% CI 0.702–0.712) for the TyG-WhtR indices.

**Table 2:** Associations between IR-related indices and the risk of pan-cancer incidence

	Model 1		Model 2		Model 3		Model 4		Model 5	
	HR (95% CI)	*P*-value	HR (95% CI)	*P*-value	HR (95% CI)	*P*-value	HR (95% CI)	*P*-value	HR (95% CI)	*P*-value
TyG										
Q1 (0–25%)	Reference		Reference		Reference		Reference		Reference	
Q2 (25–50%)	1.14 (1.11, 1.17)	< 0.001	0.97 (0.94, 0.99)	0.008	0.96 (0.93, 0.98)	0.001	0.96 (0.93, 0.98)	0.001	0.96 (0.94, 0.98)	0.002
Q3 (50–75%)	1.25 (1.22, 1.28)	< 0.001	1.00 (0.98, 1.03)	0.980	0.99 (0.96, 1.01)	0.295	0.99 (0.96, 1.01)	0.275	0.99 (0.96, 1.01)	0.300
Q4 (75–100%)	1.28 (1.25, 1.31)	< 0.001	1.01 (0.98, 1.03)	0.649	0.98 (0.96, 1.01)	0.151	0.98 (0.95, 1.01)	0.138	0.98 (0.96, 1.01)	0.154
TyG-BMI										
Q1 (0–25%)	Reference		Reference		Reference		Reference		Reference	
Q2 (25–50%)	1.15 (1.12, 1.18)	< 0.001	0.99 (0.97, 1.02)	0.470	0.99 (0.96, 1.01)	0.277	0.98 (0.96, 1.01)	0.230	0.98 (0.96, 1.01)	0.237
Q3 (50–75%)	1.24 (1.21, 1.27)	< 0.001	1.02 (1.00, 1.05)	0.071	1.01 (0.99, 1.04)	0.276	1.01 (0.99, 1.04)	0.403	1.01 (0.99, 1.04)	0.397
Q4 (75–100%)	1.24 (1.21, 1.28)	< 0.001	1.07 (1.04, 1.10)	< 0.001	1.05 (1.02, 1.08)	< 0.001	1.05 (1.02, 1.07)	0.001	1.05 (1.02, 1.07)	0.001
TyG-WC										
Q1 (0–25%)	Reference		Reference		Reference		Reference		Reference	
Q2 (25–50%)	1.24 (1.21, 1.27)	< 0.001	1.04 (1.01, 1.07)	0.006	1.03 (1.00, 1.06)	0.027	1.03 (1.00, 1.06)	0.039	1.03 (1.00, 1.06)	0.038
Q3 (50–75%)	1.38 (1.35, 1.42)	< 0.001	1.07 (1.04, 1.10)	< 0.001	1.06 (1.03, 1.09)	< 0.001	1.06 (1.03, 1.09)	< 0.001	1.06 (1.03, 1.09)	< 0.001
Q4 (75–100%)	1.51 (1.47, 1.55)	< 0.001	1.14 (1.11, 1.18)	< 0.001	1.12 (1.09, 1.15)	< 0.001	1.11 (1.08, 1.15)	< 0.001	1.11 (1.08, 1.15)	< 0.001
TyG-WHtR										
Q1 (0–25%)	Reference		Reference		Reference		Reference		Reference	
Q2 (25–50%)	1.22 (1.19, 1.25)	< 0.001	1.01 (0.98, 1.03)	0.694	1.00 (0.97, 1.03)	0.896	1.00 (0.97, 1.02)	0.821	1.00 (0.97, 1.02)	0.824
Q3 (50–75%)	1.34 (1.31, 1.38)	< 0.001	1.02 (1.00, 1.05)	0.078	1.01 (0.98, 1.04)	0.446	1.01 (0.98, 1.04)	0.551	1.01 (0.98, 1.04)	0.549
Q4 (75–100%)	1.45 (1.41, 1.48)	< 0.001	1.08 (1.05, 1.11)	< 0.001	1.06 (1.03, 1.09)	< 0.001	1.05 (1.02, 1.08)	< 0.001	1.05 (1.02, 1.08)	< 0.001

Model 1 was the raw model without adjustment

Model 2 was adjusted for age, sex, Townsend index, qualification, and ethnicity

Model 3 was further adjusted for recruitment center, alcohol consumption, smoking status, diabetes and hypertension history on the basis of Model 2

Model 4 was further adjusted for anticoagulant history and family history of cancer on the basis of Model 3

Model 5 was further adjusted for CVD, stroke, and heart failure history on the basis of Model 4

*HR* hazard ratio, *CI* confidence interval, *TyG* triglyceride‒glucose index, *BMI* body mass index, *WC* waist circumference, *WHtR* weight‒to‒height ratio

**Table 3:** Associations between IR-related indices and the risk of pan-cancer specific mortality

	Model 1		Model 2		Model 3		Model 4		Model 5	
	HR (95% CI)	*P*-value	HR (95% CI)	*P*-value	HR (95% CI)	*P*-value	HR (95% CI)	*P*-value	HR (95% CI)	*P*-value
TyG										
Q1 (0–25%)	Reference		Reference		Reference		Reference		Reference	
Q2 (25–50%)	1.23 (1.17, 1.31)	< 0.001	1.01 (0.95, 1.07)	0.780	0.99 (0.93, 1.04)	0.633	0.99 (0.93, 1.05)	0.675	0.99 (0.93, 1.05)	0.720
Q3 (50–75%)	1.44 (1.36, 1.52)	< 0.001	1.09 (1.03, 1.16)	0.002	1.05 (0.99, 1.11)	0.109	1.05 (0.99, 1.11)	0.122	1.05 (0.99, 1.11)	0.101
Q4 (75–100%)	1.62 (1.54, 1.71)	< 0.001	1.20 (1.14, 1.27)	< 0.001	1.10 (1.04, 1.16)	0.001	1.10 (1.04, 1.16)	0.001	1.10 (1.04, 1.16)	0.001
TyG-BMI										
Q1 (0–25%)	Reference		Reference		Reference		Reference		Reference	
Q2 (25–50%)	1.23 (1.16, 1.30)	< 0.001	1.02 (0.96, 1.08)	0.578	1.00 (0.95, 1.06)	0.877	1.00 (0.95, 1.06)	0.881	1.01 (0.95, 1.06)	0.859
Q3 (50–75%)	1.42 (1.34, 1.50)	< 0.001	1.12 (1.06, 1.18)	< 0.001	1.08 (1.02, 1.15)	0.006	1.08 (1.02, 1.14)	0.010	1.08 (1.02, 1.14)	0.010
Q4 (75–100%)	1.61 (1.52, 1.70)	< 0.001	1.31 (1.24, 1.39)	< 0.001	1.23 (1.16, 1.30)	< 0.001	1.22 (1.15, 1.30)	< 0.001	1.22(1.15,1.29)	< 0.001
TyG-WC										
Q1 (0–25%)	Reference		Reference		Reference		Reference		Reference	
Q2 (25–50%)	1.34 (1.27, 1.43)	< 0.001	1.08 (1.02, 1.15)	0.008	1.07 (1.00, 1.13)	0.038	1.07 (1.00, 1.13)	0.036	1.07 (1.00, 1.13)	0.035
Q3 (50–75%)	1.55 (1.46, 1.64)	< 0.001	1.15(1.08, 1.22)	< 0.001	1.11 (1.04, 1.18)	0.001	1.10 (1.04, 1.17)	0.002	1.10 (1.04, 1.17)	0.002
Q4 (75–100%)	2.07 (1.96, 2.18)	< 0.001	1.48 (1.40, 1.58)	< 0.001	1.37 (1.29, 1.46)	< 0.001	1.37 (1.28, 1.46)	< 0.001	1.37 (1.28, 1.46)	< 0.001
TyG-WHtR										
Q1 (0–25%)	Reference		Reference		Reference		Reference		Reference	
Q2 (25–50%)	1.29(1.21, 1.37)	< 0.001	1.01 (0.95, 1.07)	0.734	0.99 (0.93, 1.06)	0.801	0.99 (0.93, 1.06)	0.801	0.99 (0.93, 1.06)	0.809
Q3 (50–75%)	1.56 (1.47, 1.66)	< 0.001	1.11 (1.05, 1.18)	< 0.001	1.07 (1.01, 1.14)	0.022	1.07 (1.01, 1.14)	0.031	1.07 (1.01, 1.14)	0.030
Q4 (75–100%)	1.99 (1.88, 2.10)	< 0.001	1.36 (1.29, 1.45)	< 0.001	1.26(1.18, 1.34)	< 0.001	1.25(1.18, 1.33)	< 0.001	1.25(1.18, 1.33)	< 0.001

Model 1 was the raw model without adjustment

Model 2 was adjusted for age, sex, Townsend index, qualification, and ethnicity

Model 3 was further adjusted for recruitment center, alcohol consumption, smoking status, diabetes and hypertension history on the basis of Model 2

Model 4 was further adjusted for anticoagulant history and family history of cancer on the basis of Model 3

Model 5 was further adjusted for CVD, stroke, and heart failure history on the basis of Model 4

*HR* hazard ratio, *CI* confidence interval, *TyG* triglyceride‒glucose index, *BMI* body mass index, *WC* waist circumference, *WHtR* weight‒to‒height ratio

### Subgroup analyses

To evaluate potential effect modifications, we conducted detailed subgroup analyses to examine the interactions between covariates and IR-related indices across pan-cancer incidence and pan-cancer-specific mortality. As detailed in Supplementary Tables 1–4, significant interactions were observed between all IR-related indices and factors, including sex, smoking status, diabetes history, hypertension history, and the Townsend index, for pan-cancer incidence (p for interaction < 0.05). Anticoagulant history was significantly associated with TyG and TyG-WHtR, whereas education level was significantly associated with TyG-BMI, TyG-WC, and TyG-WHtR (p for interaction < 0.05). Additionally, we found an interaction between TyG-WC and TyG-WHtR, stratified by age or alcohol consumption (p for interaction < 0.05).

Similarly, we observed that the relationships between TyG, TyG-BMI, TyG-WC, and TyG-WHtR and pan-cancer-specific death risk were influenced by smoking status, diabetes history, and hypertension history (P for interaction < 0.05). In addition, other interaction patterns were found: age and sex interacted with TyG, alcohol consumption with TyG-BMI, TyG-WC, and TyG-WHtR; anticoagulant history with TyG, TyG-WC, and TyG-WHtR; education level with TyG-BMI; and TyG-WHtR (P for interaction < 0.05). The detailed results are presented in Supplementary Tables 5–8.

### Sensitivity analysis

Sensitivity analysis excluding participants who experienced pan-cancer events within the 1-year and 2 periods prior to follow-up did not alter the robustness of the associations (Supplementary Tables 9–10). In fully adjusted Cox models (Model 5), except for TyG, the highest quartile of the other indices remained associated with significantly elevated pan-cancer incidence and pan-cancer-specific mortality risk compared with Q1(all *p* < 0.001, Supplementary Table 11–12), whereas TyG failed to stratify pan-cancer incidence (*p* > 0.05, Supplementary Table 11–12). Notably, TyG-WC continued to exhibit the strongest association with both cancer incidence and mortality, with HRs progressively increasing from Q2 to Q4 (all *p* < 0.05 vs. Q1; Supplementary Tables 11–12). Additionally, before the above sensitivity analyses, we conducted biological plausibility checks of height, weight, and waist circumference. Participants meeting any of the following criteria were excluded before subsequent analyses: (1) Height < 120 cm or > 210 cm, (2) Weight < 30 kg or > 200 kg, (3) Body mass index < 12 kg/m^2^ or > 60 kg/m^2^, (4) Waist circumference < 50 cm or > 180 cm.

## Discussion

In this large-scale, prospective cohort study based on the UK Biobank, we evaluated, for the first time, the associations of four IR-related indices—TyG, TyG-BMI, TyG-WC, and TyG-WHtR—with both pan-cancer incidence and pan-cancer-specific mortality. Our findings indicate that these IR-related indices collectively show significant associations with pan-cancer incidence and mortality, suggesting that they may serve as surrogate markers with potential clinical utility. Besides, the findings demonstrated a certain level of value for medical institutions of tropical and near-tropical areas. Metabolic diseases, including IR and obesity, are escalating globally, particularly in tropical and low-to middle-income countries [44–46]. Most tropical and subtropical regions—except a limited number of countries and areas—are still classified as developing [47]. Healthcare systems in these regions often face challenges due to insufficient screening for common chronic illnesses like hypertension, diabetes, and cancers [48–51]. Consequently, mortality associated with these diseases remains high, mainly due to relatively limited healthcare resources and constrained funding for medical services [52–54].

In cancer screening, much current research focuses on developing novel biomarkers with greater specificity. However, both existing and newly developed tumor markers often pose a substantial financial burden for public healthcare institutions and populations; therefore, they are not well-suited to many tropical and near-tropical regions [55]. In contrast, the TyG index and its derivatives are calculated from routine parameters such as blood glucose, lipid profiles, and basic physical measurements. These are far more cost-effective than dedicated tumor markers. Notably, many tropical and subtropical regions are currently strengthening their screening systems for common chronic diseases [56, 57]. The TyG index and its relevant indices can be seamlessly integrated into these ongoing efforts—such as those targeting diabetes and dyslipidemia—without necessitating a separate, standalone cancer screening infrastructure. This offers a dual advantage: it not only serves as a potential indicator of cancer risk but also avoids additional economic burdens. Furthermore, it may act as a preliminary filter before more costly tumor-marker-based screening, thereby reducing unnecessary tests and associated costs. On the other hand, although our study utilized data from a high-income country, the study population was racially diverse, including individuals of African and Asian descent, in addition to those of White ethnicity. Subgroup analyses indicated that the predictive performance of the TyG-related index for cancer risk was generally consistent across White and non-White subgroups, suggesting its potential relevance across different ethnicities. However, we fully agree that large-scale clinical studies conducted within tropical populations are needed to confirm the predictive utility of the TyG index and its derivatives in those settings.

RCS analyses revealed that higher levels of most IR-related indices were associated with increased pan-cancer incidence. Besides, these indices also demonstrated a significant positive association with cancer-specific mortality in the cohort, consistent with prior studies [58–60].The Kaplan‒Meier curves demonstrated a consistent pattern across all four IR-related indices: participants in the Q2-Q4 category exhibited elevated cumulative hazards for both pan-cancer incidence and cancer-specific mortality compared to those in the Q1 category (all log-rank *P* < 0.001; Figs. 4 and 5), consistent with findings from recent studies [33, 34, 59]. Several mechanisms may help explain the effects of IR on cancer development. IR induces metabolic disturbances, including obesity, elevated insulin levels, hyperglycemia, and dyslipidemia, which, in turn, trigger inflammatory responses and oxidative stress. The increased release of pro-inflammatory cytokines and generation of reactive oxygen species contribute to the initiation and progression of cancer [30, 31, 61, 62]. Hyperinsulinemia may also facilitate cancer development. Insulin contributes to oncogenesis not only by abnormally activating multiple signaling cascades to promote growth factor-dependent proliferation but also by enhancing IGF-I bioactivity, consequently increasing mitogenic and anti-apoptotic signals that collectively foster tumorigenesis [28, 29]. In addition, cancer has long been viewed as a metabolic disease, with metabolic reprogramming recognized as a key requirement for tumor initiation and progression [63]. Metabolite imbalance resulting from metabolic disorders not only alters immune cell phenotypes to promote oncogenic transformation but may also directly modulate oncogenesis by altering metabolite profiles [5]. Accumulating evidence indicates that metabolic syndrome is closely associated with various precancerous conditions—such as liver fibrosis, colorectal adenomatous polyps, and oral potentially malignant disorders—as well as with the subsequent development of overt malignancies [64–66]. This supports the concept that metabolic syndrome may act as a key promoter throughout the continuum from normal tissue to precancerous lesions and finally to invasive cancer. These findings underscore the need for researchers and clinicians to address this complex multisystem condition with greater vigilance and proactive management.

Furthermore, obesity has been established as a significant risk factor for cancer development. The International Agency for Research on Cancer has recognized overweight and obesity as conditions linked to heightened cancer risk [67]. This association has been consistently validated across epidemiological studies, which confirm that elevated BMI is a significant predictor of cancer development [68, 69]. Compared to TyG, TyG-BMI, TyG-WC, and TyG-WHtR incorporate measures of obesity. Abdominal obesity, especially visceral fat, elevates levels of inflammatory factors and fatty acids, which, in turn, aggravate IR [70]. Therefore, by incorporating adiposity metrics, composite indices such as TyG-BMI, TyG-WC, and TyG-WHtR can, in many instances, yield more reliable assessments of IR and disease risk than the standard TyG index, a conclusion supported by a growing body of evidence [71–75]. These results were consistent with our findings. After adjusting for various covariates potentially associated with cancer risk, only the TyG-WC index demonstrated a favorable gradient: as the quartile increased from Q1 to Q4, the pan-cancer risk progressively rose (HR > 1, *P* < 0.05). In contrast, for the other three indices, only the Q4 group showed a statistically significant difference in pan-cancer risk compared to the Q1 reference group after adjustment (HR > 1, *P* < 0.05). Regarding pan-cancer-specific mortality, significant differences were observed for all quartiles (Q2-Q4) of the TyG-WC index compared to the reference group, suggesting a dose–response relationship between increasing TyG-WC levels and mortality risk. A similar trend was observed for TyG-WHtR and TyG-BMI, but only from the Q3 quartile onward. After adjustment, the TyG index demonstrated the weakest association for both pan-cancer incidence and mortality among the four indices. Notably, the TyG index showed no statistically significant association with pan-cancer risk, consistent with findings from two recent cohort studies [27, 76]. These findings collectively suggest that TyG-WC may be a superior risk indicator for both pan-cancer incidence and pan-cancer-specific mortality. This finding aligns with existing evidence, as TyG-WC's independent effect has also been observed in cancer-specific mortality among prediabetic patients and in the risk of hyperuricemia among hypertensive populations [59, 77]. As mentioned in many studies, WHtR is less influenced by factors such as sex, ethnicity, and height, often providing a more accurate reflection of individual adiposity and demonstrating better predictive power for CVD risk than WC alone [78, 79]. However, in the context of cancer risk, accumulating evidence suggests that abdominal fat accumulation may be a significant risk factor for various malignancies, including lung, breast, and cervical cancer [80–82]. In this regard, WC is a more direct and unmediated indicator of abdominal adiposity than WHtR. This distinction may explain why the TyG-WC demonstrated better performance for both pan-cancer incidence and specific mortality in our study than the other indices. However, it should not be neglected that the C-index of the associations between TyG-WC and pan-cancer incidence or mortality were 0.650 and 0.707, respectively. The modest C-index indicated that absolute predictive performance of TyG-WC is still moderate, and the index should be viewed as a complementary risk marker rather than a stand-alone screening tool.

Our results of sensitivity analyses confirmed the association between most IR-related indices (excluding TyG) and pan-cancer risk and mortality. TyG-WC consistently demonstrated the strongest and graded association for both cancer incidence and mortality. Besides, the results of subgroup analyses showed that the association between IR-related indices and pan-cancer incidence was more significant in a specific population, namely low-income females with no smoking history and without chronic diseases, with a similar pattern observed for cancer-specific mortality, suggesting the potential utility of these indices across diverse demographic groups. The following potential mechanisms may explain population differences. For women, IR and hyperinsulinemia lower Sex Hormone-Binding Globulin levels, increasing free sex hormones, while IGF-1 interacts with insulin signaling and hormone receptors, collectively promoting the development of hormone-sensitive tumors in women, such as breast and endometrial cancer [29, 83]. Both smoking and IR can induce oxidative stress. In smokers, oxidative damage may dominate tumorigenic mechanisms, whereas in non-smokers, IR-driven hyperinsulinemia and IGF signaling play a more independent role [84, 85]. In people with no chronic diseases, IR could be an earlier stage with sustained compensatory hyperinsulinemia, allowing pro-tumorigenic pathways (high insulin/IGF-1, proliferation, anti-apoptosis, low-grade inflammation, oxidative stress) to act more directly. In contrast, in those with chronic diseases, existing metabolic disturbances, organ damage, and treatments may obscure or dilute the independent effect of IR on cancer risk [86]. For low-income individuals, poor economic conditions may strengthen the link between IR and cancer through promoting chronic inflammation, adverse lifestyle factors, and cumulative metabolic stress, increasing the pro-tumorigenic effects caused by IR [87, 88]. A study carried out by Spanish researchers revealed that brown adipose tissue activity could be sensitive to temperature changes, indicating that long-term situating in warmer climates may be associated with a higher risk of diabetes and IR [89]. This observation is supported by additional studies reporting a close relationship among ambient temperature, diabetes prevalence, and IR [90, 91]. Therefore, compared to temperate regions, the TyG index and its derived indices may hold even broader promise in tropical and near-tropical regions. They not only serve as practical indicators of IR but also as accessible risk markers of pan-cancer risk and associated mortality. In particular, the robustness of the TyG-WC index across models with extensive covariate adjustment, subgroup analyses, and sensitivity analyses in this study highlights its generalizability. The TyG-WC index can be incorporated into chronic disease screening programs, requiring minimal additional testing and offering a low-cost indication of cancer risk. It shows promise as an initial screening tool and risk marker, mainly benefiting low-income populations and regions with limited healthcare resources. Risk cut-off values for TyG-WC, such as the 739.624 identified in this study, may be used to stratify populations into high- and low-risk groups for cancer, thereby facilitating targeted health management and further medical evaluation.

Nonetheless, our study has several limitations. Primarily, IR-related indices were calculated at baseline and treated as static; changes in behavior or clinical metrics over time were not considered, making it difficult to infer the dynamic impact of fluctuations in IR-related indices on pan-cancer incidence and mortality. Future studies incorporating repeated assessments could help elucidate the effects of longitudinal improvements or deterioration in health. Then, the number of deaths related to cancer was limited; therefore, the actual association of IR-related indices with pan-cancer-specific mortality still requires validation. Next, despite comprehensive adjustment for numerous covariates, our findings remain susceptible to residual confounding by unmeasured factors, including lifestyle characteristics (e.g., physical activity, diet) or metabolic-related interventions (e.g., lipid-lowering or antidiabetic medications), and potential reverse causality cannot be ruled out. Besides, we adopted complete-case strategy during constructing the analysis cohort. The exclusion of participants due to missing covariates may introduce selection bias. What’s more, this study was based exclusively on data from the UK Biobank. Given that the cohort is predominantly White, the generalizability of our findings to other ethnicities and regions is limited, and external validation in more diverse populations is warranted. Finally, as with all observational studies, causality cannot be inferred from the observed associations.

## Conclusion

This prospective study identified significant associations between IR-related indices, particularly TyG-WC, and the incidence and prognosis of cancer. Moreover, TyG-WC demonstrated stronger associations with both pan-cancer incidence and pan-cancer-specific mortality than other IR-related indices. These findings underscore the potential utility of incorporating IR-related indices to prevent cancer and screen high-risk populations.

## Supplementary Information

Supplementary material 1.

Supplementary material 2.

## Data Availability

UK Biobank data are available through application to the database https://www.ukbiobank.ac.uk/. The data that support the findings of this study are available from UK Biobank but restrictions apply to the availability of these data, which were used under license for the current study, and so are not publicly available. Data are however available from the authors upon reasonable request and with permission of UK Biobank.

## Abbreviations

IR: Insulin resistance
CVD: Cardiovascular disease
NAFLD: Non-alcoholic fatty liver disease
HOMA-IR: Homeostasis Model Assessment of Insulin Resistance
TyG: Triglyceride-glucose
TG: Triglyceride
IGF-1: Insulin-like Growth Factor 1
BMI: Body mass index
WC: Waist circumference
WHtR: Waist-to-height ratio
STROBE: Strengthening the Reporting of Observational Studies in Epidemiology
HDL-C: High-density lipoprotein cholesterol
IQR: Interquartile range
HR: Hazard ratio
CI: Confidence interval
RCS: Restricted cubic spline
