# Supplementary material for: Comprehensive evaluation of the prevalent insulin resistance indices for pan-cancer incidence and mortality prediction
Source: Trop Med Health. 2025 Dec 19;54:9. doi: 10.1186/s41182-025-00884-5 (PMC12781529; doi:10.1186/s41182-025-00884-5)
Supplement: Supplementary file 1 — Supplementary material 1. [file 41182_2025_884_MOESM1_ESM.docx]

**Supplementary Table 1. Subgroup analysis of TyG index for pan-cancer incidence**

| **Variable** | **Count** | **Percent** | **OR** | **Lower** | **Upper** | ***P*-value** | ***P* for interaction** |
| --- | --- | --- | --- | --- | --- | --- | --- |
| Age |  |  |  |  |  |  | 0.571 |
| <60 | 209168 | 62.8 | 1.09 | 1.07 | 1.12 | <0.001 |  |
| ≥60 | 124129 | 37.2 | 1.08 | 1.06 | 1.11 | <0.001 |  |
| Sex |  |  |  |  |  |  | <0.001 |
| Female | 179063 | 53.7 | 1.26 | 1.24 | 1.29 | <0.001 |  |
| Male | 154234 | 46.3 | 1.01 | 0.99 | 1.03 | 0.508 |  |
| Education level |  |  |  |  |  |  | 0.692 |
| College or University degree | 132016 | 39.6 | 1.16 | 1.14 | 1.19 | <0.001 |  |
| Less than a college degree | 201281 | 60.4 | 1.17 | 1.15 | 1.19 | <0.001 |  |
| Smoking status |  |  |  |  |  |  | <0.001 |
| No | 301071 | 90.3 | 1.18 | 1.16 | 1.20 | <0.001 |  |
| Yes | 32226 | 9.7 | 1.08 | 1.03 | 1.13 | 0.001 |  |
| Alcohol consumption |  |  |  |  |  |  | 0.638 |
| No | 22815 | 6.8 | 1.16 | 1.09 | 1.23 | <0.001 |  |
| Yes | 310482 | 93.2 | 1.17 | 1.16 | 1.19 | <0.001 |  |
| Ethnicity |  |  |  |  |  |  | 0.664 |
| Non-white | 18,789 | 5.6 | 1.16 | 1.10 | 1.22 | <0.001 |  |
| White | 314,508 | 94.4 | 1.17 | 1.15 | 1.19 | <0.001 |  |
| Diabetes history |  |  |  |  |  |  | <0.001 |
| No | 304274 | 91.3 | 1.15 | 1.13 | 1.17 | <0.001 |  |
| Yes | 29023 | 8.7 | 0.93 | 0.89 | 0.96 | <0.001 |  |
| Hypertension history |  |  |  |  |  |  | <0.001 |
| No | 160048 | 48.0 | 1.16 | 1.13 | 1.19 | <0.001 |  |
| Yes | 173249 | 52.0 | 1.07 | 1.04 | 1.09 | <0.001 |  |
| Townsend index |  |  |  |  |  |  | 0.029 |
| Q1 | 89609 | 26.9 | 1.21 | 1.18 | 1.25 | <0.001 |  |
| Q2 | 86427 | 25.9 | 1.18 | 1.15 | 1.22 | <0.001 |  |
| Q3 | 82931 | 24.9 | 1.16 | 1.13 | 1.20 | <0.001 |  |
| Q4 | 74330 | 22.3 | 1.14 | 1.10 | 1.18 | <0.001 |  |
| Center |  |  |  |  |  |  | 0.560 |
| England | 295225 | 88.6 | 1.17 | 1.16 | 1.19 | <0.001 |  |
| Scotland | 23627 | 7.1 | 1.17 | 1.10 | 1.24 | <0.001 |  |
| Wales | 14445 | 4.3 | 1.13 | 1.05 | 1.21 | 0.001 |  |
| Family cancer history |  |  |  |  |  |  | 0.317 |
| No | 219211 | 65.8 | 1.18 | 1.16 | 1.20 | <0.001 |  |
| Yes | 114086 | 34.2 | 1.16 | 1.13 | 1.19 | <0.001 |  |
| Anticoagulant history |  |  |  |  |  |  | <0.001 |
| No | 289019 | 87.5 | 1.17 | 1.15 | 1.19 | <0.001 |  |
| Yes | 41313 | 12.5 | 1.06 | 1.02 | 1.10 | 0.001 |  |

Abbreviations: OR: Odds Ratio.

**Supplementary Table 2.** **Subgroup analysis of TyG-BMI index for pan-cancer incidence**

| **Variable** | **Count** | **Percent** | **OR** | **Lower** | **Upper** | ***P*-value** | ***P* for interaction** |
| --- | --- | --- | --- | --- | --- | --- | --- |
| Age |  |  |  |  |  |  | 0.099 |
| <60 | 209168 | 62.8 | 1.11 | 1.08 | 1.13 | <0.001 |  |
| ≥60 | 124129 | 37.2 | 1.14 | 1.11 | 1.17 | <0.001 |  |
| Sex |  |  |  |  |  |  | <0.001 |
| Female | 179063 | 53.7 | 1.19 | 1.16 | 1.22 | <0.001 |  |
| Male | 154234 | 46.3 | 1.03 | 1.00 | 1.06 | 0.035 |  |
| Education level |  |  |  |  |  |  | 0.013 |
| College or University degree | 132016 | 39.6 | 1.18 | 1.15 | 1.22 | <0.001 |  |
| Less than a college degree | 201281 | 60.4 | 1.13 | 1.11 | 1.15 | <0.001 |  |
| Smoking status |  |  |  |  |  |  | <0.001 |
| No | 301071 | 90.3 | 1.17 | 1.15 | 1.20 | <0.001 |  |
| Yes | 32226 | 9.7 | 1.01 | 0.96 | 1.07 | 0.74 |  |
| Alcohol consumption |  |  |  |  |  |  | 0.281 |
| No | 22815 | 6.8 | 1.12 | 1.06 | 1.19 | <0.001 |  |
| Yes | 310482 | 93.2 | 1.16 | 1.14 | 1.18 | <0.001 |  |
| Ethnicity |  |  |  |  |  |  | 0.752 |
| Non-white | 18,789 | 5.6 | 1.16 | 1.10 | 1.24 | <0.001 |  |
| White | 314,508 | 94.4 | 1.15 | 1.13 | 1.17 | <0.001 |  |
| Diabetes history |  |  |  |  |  |  | <0.001 |
| No | 304274 | 91.3 | 1.11 | 1.09 | 1.14 | <0.001 |  |
| Yes | 29023 | 8.7 | 0.86 | 0.83 | 0.9 | <0.001 |  |
| Hypertension history |  |  |  |  |  |  | <0.001 |
| No | 160048 | 48.0 | 1.10 | 1.06 | 1.13 | <0.001 |  |
| Yes | 173249 | 52.0 | 1.02 | 1.00 | 1.05 | 0.032 |  |
| Townsend index |  |  |  |  |  |  | 0.001 |
| Q1 | 89609 | 26.9 | 1.22 | 1.18 | 1.26 | <0.001 |  |
| Q2 | 86427 | 25.9 | 1.17 | 1.13 | 1.21 | <0.001 |  |
| Q3 | 82931 | 24.9 | 1.18 | 1.14 | 1.22 | <0.001 |  |
| Q4 | 74330 | 22.3 | 1.10 | 1.06 | 1.14 | <0.001 |  |
| Center |  |  |  |  |  |  | 0.084 |
| England | 295225 | 88.6 | 1.15 | 1.13 | 1.17 | <0.001 |  |
| Scotland | 23627 | 7.1 | 1.24 | 1.16 | 1.33 | <0.001 |  |
| Wales | 14445 | 4.3 | 1.10 | 1.02 | 1.20 | 0.016 |  |
| Family cancer history |  |  |  |  |  |  | 0.612 |
| No | 219211 | 65.8 | 1.16 | 1.13 | 1.18 | <0.001 |  |
| Yes | 114086 | 34.2 | 1.15 | 1.11 | 1.18 | <0.001 |  |
| Anticoagulant history |  |  |  |  |  |  | 0.309 |
| No | 289019 | 87.5 | 1.12 | 1.10 | 1.14 | <0.001 |  |
| Yes | 41313 | 12.5 | 1.10 | 1.05 | 1.14 | <0.001 |  |

Abbreviations: OR: Odds Ratio.

**Supplementary Table 3.** **Subgroup analysis of TyG-WC index for pan-cancer incidence**

| **Variable** | **Count** | **Percent** | **OR** | **Lower** | **Upper** | ***P*-value** | ***P* for interaction** |
| --- | --- | --- | --- | --- | --- | --- | --- |
| Age |  |  |  |  |  |  | <0.001 |
| <60 | 209168 | 62.8 | 1.11 | 1.09 | 1.13 | <0.001 |  |
| ≥60 | 124129 | 37.2 | 1.23 | 1.21 | 1.25 | <0.001 |  |
| Sex |  |  |  |  |  |  | <0.001 |
| Female | 179063 | 53.7 | 1.21 | 1.18 | 1.23 | <0.001 |  |
| Male | 154234 | 46.3 | 1.11 | 1.09 | 1.13 | <0.001 |  |
| Education level |  |  |  |  |  |  | 0.021 |
| College or University degree | 132016 | 39.6 | 1.24 | 1.21 | 1.26 | <0.001 |  |
| Less than a college degree | 201281 | 60.4 | 1.20 | 1.19 | 1.22 | <0.001 |  |
| Smoking status |  |  |  |  |  |  | <0.001 |
| No | 301071 | 90.3 | 1.23 | 1.21 | 1.24 | <0.001 |  |
| Yes | 32226 | 9.7 | 1.14 | 1.09 | 1.18 | <0.001 |  |
| Alcohol consumption |  |  |  |  |  |  | 0.019 |
| No | 22815 | 6.8 | 1.16 | 1.11 | 1.21 | <0.001 |  |
| Yes | 310482 | 93.2 | 1.22 | 1.21 | 1.24 | <0.001 |  |
| Ethnicity |  |  |  |  |  |  | 0.569 |
| Non-white | 18,789 | 5.6 | 1.23 | 1.18 | 1.28 | <0.001 |  |
| White | 314,508 | 94.4 | 1.22 | 1.20 | 1.23 | <0.001 |  |
| Diabetes history |  |  |  |  |  |  | <0.001 |
| No | 304274 | 91.3 | 1.20 | 1.19 | 1.22 | <0.001 |  |
| Yes | 29023 | 8.7 | 0.97 | 0.94 | 1.01 | 0.106 |  |
| Hypertension history |  |  |  |  |  |  | 0.001 |
| No | 160048 | 48.0 | 1.18 | 1.16 | 1.20 | <0.001 |  |
| Yes | 173249 | 52.0 | 1.13 | 1.11 | 1.15 | <0.001 |  |
| Townsend index |  |  |  |  |  |  | <0.001 |
| Q1 | 89609 | 26.9 | 1.28 | 1.25 | 1.30 | <0.001 |  |
| Q2 | 86427 | 25.9 | 1.24 | 1.21 | 1.27 | <0.001 |  |
| Q3 | 82931 | 24.9 | 1.21 | 1.18 | 1.24 | <0.001 |  |
| Q4 | 74330 | 22.3 | 1.17 | 1.14 | 1.19 | <0.001 |  |
| Center |  |  |  |  |  |  | 0.194 |
| England | 295225 | 88.6 | 1.22 | 1.20 | 1.23 | <0.001 |  |
| Scotland | 23627 | 7.1 | 1.27 | 1.21 | 1.33 | <0.001 |  |
| Wales | 14445 | 4.3 | 1.19 | 1.12 | 1.25 | <0.001 |  |
| Family cancer history |  |  |  |  |  |  | 0.916 |
| No | 219211 | 65.8 | 1.22 | 1.20 | 1.24 | <0.001 |  |
| Yes | 114086 | 34.2 | 1.22 | 1.19 | 1.24 | <0.001 |  |
| Anticoagulant history |  |  |  |  |  |  | 0.122 |
| No | 289019 | 87.5 | 1.19 | 1.18 | 1.21 | <0.001 |  |
| Yes | 41313 | 12.5 | 1.16 | 1.13 | 1.20 | <0.001 |  |

Abbreviations: OR: Odds Ratio.

**Supplementary Table 4. Subgroup analysis of TyG-WHtR index for pan-cancer incidence**

| **Variable** | **Count** | **Percent** | **OR** | **Lower** | **Upper** | ***P*-value** | ***P* for interaction** |
| --- | --- | --- | --- | --- | --- | --- | --- |
| Age |  |  |  |  |  |  | 0.021 |
| <60 | 209168 | 62.8 | 1.09 | 1.07 | 1.11 | <0.001 |  |
| ≥60 | 124129 | 37.2 | 1.12 | 1.10 | 1.13 | <0.001 |  |
| Sex |  |  |  |  |  |  | <0.001 |
| Female | 179063 | 53.7 | 1.15 | 1.14 | 1.17 | <0.001 |  |
| Male | 154234 | 46.3 | 1.11 | 1.09 | 1.13 | <0.001 |  |
| Education level |  |  |  |  |  |  | 0.023 |
| College or University degree | 132016 | 39.6 | 1.18 | 1.16 | 1.20 | <0.001 |  |
| Less than a college degree | 201281 | 60.4 | 1.15 | 1.14 | 1.17 | <0.001 |  |
| Smoking status |  |  |  |  |  |  | 0.007 |
| No | 301071 | 90.3 | 1.17 | 1.16 | 1.19 | <0.001 |  |
| Yes | 32226 | 9.7 | 1.12 | 1.08 | 1.16 | <0.001 |  |
| Alcohol consumption |  |  |  |  |  |  | 0.024 |
| No | 22815 | 6.8 | 1.12 | 1.08 | 1.16 | <0.001 |  |
| Yes | 310482 | 93.2 | 1.17 | 1.16 | 1.19 | <0.001 |  |
| Ethnicity |  |  |  |  |  |  | 0.793 |
| Non-white | 18,789 | 5.6 | 1.17 | 1.13 | 1.22 | <0.001 |  |
| White | 314,508 | 94.4 | 1.17 | 1.16 | 1.18 | <0.001 |  |
| Diabetes history |  |  |  |  |  |  | <0.001 |
| No | 304274 | 91.3 | 1.15 | 1.14 | 1.17 | <0.001 |  |
| Yes | 29023 | 8.7 | 0.95 | 0.92 | 0.98 | <0.001 |  |
| Hypertension history |  |  |  |  |  |  | <0.001 |
| No | 160048 | 48.0 | 1.14 | 1.12 | 1.16 | <0.001 |  |
| Yes | 173249 | 52.0 | 1.08 | 1.07 | 1.10 | <0.001 |  |
| Townsend index |  |  |  |  |  |  | <0.001 |
| Q1 | 89609 | 26.9 | 1.21 | 1.19 | 1.24 | <0.001 |  |
| Q2 | 86427 | 25.9 | 1.18 | 1.16 | 1.21 | <0.001 |  |
| Q3 | 82931 | 24.9 | 1.17 | 1.15 | 1.20 | <0.001 |  |
| Q4 | 74330 | 22.3 | 1.14 | 1.11 | 1.16 | <0.001 |  |
| Center |  |  |  |  |  |  | 0.048 |
| England | 295225 | 88.6 | 1.17 | 1.15 | 1.18 | <0.001 |  |
| Scotland | 23627 | 7.1 | 1.22 | 1.17 | 1.27 | <0.001 |  |
| Wales | 14445 | 4.3 | 1.13 | 1.08 | 1.19 | <0.001 |  |
| Family cancer history |  |  |  |  |  |  | 0.301 |
| No | 219211 | 65.8 | 1.17 | 1.16 | 1.19 | <0.001 |  |
| Yes | 114086 | 34.2 | 1.16 | 1.14 | 1.18 | <0.001 |  |
| Anticoagulant history |  |  |  |  |  |  | 0.022 |
| No | 289019 | 87.5 | 1.15 | 1.14 | 1.16 | <0.001 |  |
| Yes | 41313 | 12.5 | 1.11 | 1.09 | 1.14 | <0.001 |  |

Abbreviations: OR: Odds Ratio.

**Supplementary Table 5.** **Subgroup analysis of TyG index for pan-cancer specific mortality**

| **Variable** | **Count** | **Percent** | **OR** | **Lower** | **Upper** | ***P*-value** | ***P* for interaction** |
| --- | --- | --- | --- | --- | --- | --- | --- |
| Age |  |  |  |  |  |  | 0.009 |
| <60 | 208381 | 62.9 | 1.34 | 1.27 | 1.41 | <0.001 |  |
| ≥60 | 122774 | 37.1 | 1.22 | 1.17 | 1.28 | <0.001 |  |
| Sex |  |  |  |  |  |  | <0.001 |
| Female | 178100 | 53.8 | 1.53 | 1.45 | 1.61 | <0.001 |  |
| Male | 153055 | 46.2 | 1.17 | 1.12 | 1.22 | <0.001 |  |
| Education level |  |  |  |  |  |  | 0.164 |
| College or University degree | 131279 | 39.6 | 1.42 | 1.34 | 1.50 | <0.001 |  |
| Less than a college degree | 199876 | 60.4 | 1.35 | 1.3 | 1.41 | <0.001 |  |
| Smoking status |  |  |  |  |  |  | <0.001 |
| No | 299258 | 90.4 | 1.40 | 1.35 | 1.45 | <0.001 |  |
| Yes | 31897 | 9.6 | 1.16 | 1.07 | 1.25 | <0.001 |  |
| Alcohol consumption |  |  |  |  |  |  | 0.225 |
| No | 22679 | 6.8 | 1.29 | 1.16 | 1.45 | <0.001 |  |
| Yes | 308476 | 93.2 | 1.39 | 1.34 | 1.44 | <0.001 |  |
| Ethnicity |  |  |  |  |  |  | 0.930 |
| Non-white | 18,789 | 5.6 | 1.38 | 1.23 | 1.53 | <0.001 |  |
| White | 314,508 | 94.4 | 1.38 | 1.34 | 1.43 | <0.001 |  |
| Diabetes history |  |  |  |  |  |  | <0.001 |
| No | 302513 | 91.4 | 1.31 | 1.26 | 1.36 | <0.001 |  |
| Yes | 28642 | 8.6 | 1.04 | 0.97 | 1.12 | 0.256 |  |
| Hypertension history |  |  |  |  |  |  | <0.001 |
| No | 159270 | 48.1 | 1.38 | 1.30 | 1.46 | <0.001 |  |
| Yes | 171885 | 51.9 | 1.21 | 1.16 | 1.26 | <0.001 |  |
| Townsend index |  |  |  |  |  |  | 0.265 |
| Q1 | 89068 | 26.9 | 1.44 | 1.35 | 1.54 | <0.001 |  |
| Q2 | 85869 | 25.9 | 1.36 | 1.28 | 1.45 | <0.001 |  |
| Q3 | 82380 | 24.9 | 1.41 | 1.32 | 1.50 | <0.001 |  |
| Q4 | 73838 | 22.3 | 1.33 | 1.24 | 1.41 | <0.001 |  |
| Center |  |  |  |  |  |  | 0.640 |
| England | 293293 | 88.6 | 1.39 | 1.35 | 1.44 | <0.001 |  |
| Scotland | 23518 | 7.1 | 1.36 | 1.23 | 1.51 | <0.001 |  |
| Wales | 14344 | 4.3 | 1.31 | 1.13 | 1.52 | <0.001 |  |
| Family cancer history |  |  |  |  |  |  | 0.407 |
| No | 217908 | 65.8 | 1.40 | 1.34 | 1.45 | <0.001 |  |
| Yes | 113247 | 34.2 | 1.36 | 1.29 | 1.43 | <0.001 |  |
| Anticoagulant history |  |  |  |  |  |  | 0.001 |
| No | 287302 | 87.5 | 1.38 | 1.33 | 1.43 | <0.001 |  |
| Yes | 40903 | 12.5 | 1.21 | 1.12 | 1.29 | <0.001 |  |

Abbreviations: OR: Odds Ratio.

**Supplementary Table 6**. **Subgroup analysis of TyG-BMI index for pan-cancer specific mortality**

| **Variable** | **Count** | **Percent** | **OR** | **Lower** | **Upper** | ***P*-value** | ***P* for interaction** |
| --- | --- | --- | --- | --- | --- | --- | --- |
| Age |  |  |  |  |  |  | 0.551 |
| <60 | 208381 | 62.9 | 1.37 | 1.29 | 1.45 | <0.001 |  |
| ≥60 | 122774 | 37.1 | 1.34 | 1.27 | 1.41 | <0.001 |  |
| Sex |  |  |  |  |  |  | 0.056 |
| Female | 178100 | 53.8 | 1.38 | 1.32 | 1.45 | <0.001 |  |
| Male | 153055 | 46.2 | 1.29 | 1.22 | 1.36 | <0.001 |  |
| Education level |  |  |  |  |  |  | <0.001 |
| College or University degree | 131279 | 39.6 | 1.50 | 1.41 | 1.60 | <0.001 |  |
| Less than a college degree | 199876 | 60.4 | 1.30 | 1.24 | 1.36 | <0.001 |  |
| Smoking status |  |  |  |  |  |  | <0.001 |
| No | 299258 | 90.4 | 1.48 | 1.42 | 1.54 | <0.001 |  |
| Yes | 31897 | 9.6 | 0.93 | 0.85 | 1.03 | 0.162 |  |
| Alcohol consumption |  |  |  |  |  |  | 0.003 |
| No | 22679 | 6.8 | 1.17 | 1.04 | 1.31 | 0.009 |  |
| Yes | 308476 | 93.2 | 1.41 | 1.35 | 1.46 | <0.001 |  |
| Ethnicity |  |  |  |  |  |  | 0.296 |
| Non-white | 18,789 | 5.6 | 1.30 | 1.14 | 1.47 | <0.001 |  |
| White | 314,508 | 94.4 | 1.39 | 1.34 | 1.44 | <0.001 |  |
| Diabetes history |  |  |  |  |  |  | <0.001 |
| No | 302513 | 91.4 | 1.31 | 1.25 | 1.36 | <0.001 |  |
| Yes | 28642 | 8.6 | 0.92 | 0.85 | 1.00 | 0.049 |  |
| Hypertension history |  |  |  |  |  |  | 0.019 |
| No | 159270 | 48.1 | 1.30 | 1.21 | 1.40 | <0.001 |  |
| Yes | 171885 | 51.9 | 1.18 | 1.13 | 1.23 | <0.001 |  |
| Townsend index |  |  |  |  |  |  | 0.002 |
| Q1 | 89068 | 26.9 | 1.47 | 1.37 | 1.59 | <0.001 |  |
| Q2 | 85869 | 25.9 | 1.42 | 1.32 | 1.53 | <0.001 |  |
| Q3 | 82380 | 24.9 | 1.44 | 1.34 | 1.55 | <0.001 |  |
| Q4 | 73838 | 22.3 | 1.24 | 1.15 | 1.32 | <0.001 |  |
| Center |  |  |  |  |  |  | 0.575 |
| England | 293293 | 88.6 | 1.39 | 1.34 | 1.45 | <0.001 |  |
| Scotland | 23518 | 7.1 | 1.39 | 1.24 | 1.57 | <0.001 |  |
| Wales | 14344 | 4.3 | 1.28 | 1.08 | 1.50 | 0.003 |  |
| Family cancer history |  |  |  |  |  |  | 0.177 |
| No | 217908 | 65.8 | 1.41 | 1.35 | 1.47 | <0.001 |  |
| Yes | 113247 | 34.2 | 1.34 | 1.26 | 1.42 | <0.001 |  |
| Anticoagulant history |  |  |  |  |  |  | 0.157 |
| No | 287302 | 87.5 | 1.34 | 1.29 | 1.40 | <0.001 |  |
| Yes | 40903 | 12.5 | 1.26 | 1.17 | 1.36 | <0.001 |  |

Abbreviations: OR: Odds Ratio.

**Supplementary Table 7. Subgroup analysis of TyG-WC index for pan-cancer specific mortality**

| **Variable** | **Count** | **Percent** | **OR** | **Lower** | **Upper** | ***P*-value** | ***P* for interaction** |
| --- | --- | --- | --- | --- | --- | --- | --- |
| Age |  |  |  |  |  |  | 0.991 |
| <60 | 208381 | 62.9 | 1.17 | 1.15 | 1.19 | <0.001 |  |
| ≥60 | 122774 | 37.1 | 1.17 | 1.15 | 1.19 | <0.001 |  |
| Sex |  |  |  |  |  |  | 0.096 |
| Female | 178100 | 53.8 | 1.19 | 1.17 | 1.21 | <0.001 |  |
| Male | 153055 | 46.2 | 1.16 | 1.14 | 1.18 | <0.001 |  |
| Education level |  |  |  |  |  |  | 0.072 |
| College or University degree | 131279 | 39.6 | 1.21 | 1.19 | 1.24 | <0.001 |  |
| Less than a college degree | 199876 | 60.4 | 1.18 | 1.16 | 1.20 | <0.001 |  |
| Smoking status |  |  |  |  |  |  | <0.001 |
| No | 299258 | 90.4 | 1.21 | 1.20 | 1.23 | <0.001 |  |
| Yes | 31897 | 9.6 | 1.08 | 1.05 | 1.11 | <0.001 |  |
| Alcohol consumption |  |  |  |  |  |  | 0.002 |
| No | 22679 | 6.8 | 1.12 | 1.08 | 1.17 | <0.001 |  |
| Yes | 308476 | 93.2 | 1.20 | 1.19 | 1.22 | <0.001 |  |
| Ethnicity |  |  |  |  |  |  | 0.214 |
| Non-white | 18,789 | 5.6 | 1.16 | 1.11 | 1.22 | <0.001 |  |
| White | 314,508 | 94.4 | 1.20 | 1.18 | 1.21 | <0.001 |  |
| Diabetes history |  |  |  |  |  |  | <0.001 |
| No | 302513 | 91.4 | 1.18 | 1.16 | 1.20 | <0.001 |  |
| Yes | 28642 | 8.6 | 1.03 | 1.00 | 1.06 | 0.032 |  |
| Hypertension history |  |  |  |  |  |  | 0.026 |
| No | 159270 | 48.1 | 1.17 | 1.15 | 1.20 | <0.001 |  |
| Yes | 171885 | 51.9 | 1.14 | 1.12 | 1.15 | <0.001 |  |
| Townsend index |  |  |  |  |  |  | 0.066 |
| Q1 | 89068 | 26.9 | 1.21 | 1.18 | 1.24 | <0.001 |  |
| Q2 | 85869 | 25.9 | 1.21 | 1.18 | 1.24 | <0.001 |  |
| Q3 | 82380 | 24.9 | 1.20 | 1.17 | 1.23 | <0.001 |  |
| Q4 | 73838 | 22.3 | 1.16 | 1.14 | 1.19 | <0.001 |  |
| Center |  |  |  |  |  |  | 0.497 |
| England | 293293 | 88.6 | 1.20 | 1.18 | 1.21 | <0.001 |  |
| Scotland | 23518 | 7.1 | 1.21 | 1.16 | 1.26 | <0.001 |  |
| Wales | 14344 | 4.3 | 1.16 | 1.10 | 1.23 | <0.001 |  |
| Family cancer history |  |  |  |  |  |  | 0.081 |
| No | 217908 | 65.8 | 1.21 | 1.19 | 1.22 | <0.001 |  |
| Yes | 113247 | 34.2 | 1.18 | 1.16 | 1.20 | <0.001 |  |
| Anticoagulant history |  |  |  |  |  |  | 0.017 |
| No | 287302 | 87.5 | 1.18 | 1.17 | 1.20 | <0.001 |  |
| Yes | 40903 | 12.5 | 1.14 | 1.11 | 1.17 | <0.001 |  |

Abbreviations: OR: Odds Ratio.

**Supplementary Table 8. Subgroup analysis of TyG-WHtR index for pan-cancer specific mortality**

| **Variable** | **Count** | **Percent** | **OR** | **Lower** | **Upper** | ***P*-value** | ***P* for interaction** |
| --- | --- | --- | --- | --- | --- | --- | --- |
| Age |  |  |  |  |  |  | 0.109 |
| <60 | 208381 | 62.9 | 1.31 | 1.26 | 1.36 | <0.001 |  |
| ≥60 | 122774 | 37.1 | 1.26 | 1.22 | 1.30 | <0.001 |  |
| Sex |  |  |  |  |  |  | 0.975 |
| Female | 178100 | 53.8 | 1.32 | 1.28 | 1.36 | <0.001 |  |
| Male | 153055 | 46.2 | 1.32 | 1.28 | 1.37 | <0.001 |  |
| Education level |  |  |  |  |  |  | 0.009 |
| College or University degree | 131279 | 39.6 | 1.41 | 1.35 | 1.46 | <0.001 |  |
| Less than a college degree | 199876 | 60.4 | 1.32 | 1.29 | 1.36 | <0.001 |  |
| Smoking status |  |  |  |  |  |  | <0.001 |
| No | 299258 | 90.4 | 1.39 | 1.36 | 1.42 | <0.001 |  |
| Yes | 31897 | 9.6 | 1.16 | 1.09 | 1.22 | <0.001 |  |
| Alcohol consumption |  |  |  |  |  |  | 0.002 |
| No | 22679 | 6.8 | 1.22 | 1.13 | 1.31 | <0.001 |  |
| Yes | 308476 | 93.2 | 1.37 | 1.34 | 1.40 | <0.001 |  |
| Ethnicity |  |  |  |  |  |  | 0.245 |
| Non-white | 18,789 | 5.6 | 1.30 | 1.21 | 1.40 | <0.001 |  |
| White | 314,508 | 94.4 | 1.36 | 1.33 | 1.40 | <0.001 |  |
| Diabetes history |  |  |  |  |  |  | <0.001 |
| No | 302513 | 91.4 | 1.33 | 1.29 | 1.36 | <0.001 |  |
| Yes | 28642 | 8.6 | 1.03 | 0.97 | 1.08 | 0.317 |  |
| Hypertension history |  |  |  |  |  |  | 0.001 |
| No | 159270 | 48.1 | 1.34 | 1.28 | 1.39 | <0.001 |  |
| Yes | 171885 | 51.9 | 1.23 | 1.19 | 1.26 | <0.001 |  |
| Townsend index |  |  |  |  |  |  | 0.087 |
| Q1 | 89068 | 26.9 | 1.39 | 1.33 | 1.46 | <0.001 |  |
| Q2 | 85869 | 25.9 | 1.37 | 1.31 | 1.43 | <0.001 |  |
| Q3 | 82380 | 24.9 | 1.38 | 1.32 | 1.44 | <0.001 |  |
| Q4 | 73838 | 22.3 | 1.30 | 1.25 | 1.35 | <0.001 |  |
| Center |  |  |  |  |  |  | 0.636 |
| England | 293293 | 88.6 | 1.36 | 1.33 | 1.40 | <0.001 |  |
| Scotland | 23518 | 7.1 | 1.39 | 1.29 | 1.49 | <0.001 |  |
| Wales | 14344 | 4.3 | 1.31 | 1.18 | 1.44 | <0.001 |  |
| Family cancer history |  |  |  |  |  |  | 0.038 |
| No | 217908 | 65.8 | 1.38 | 1.34 | 1.42 | <0.001 |  |
| Yes | 113247 | 34.2 | 1.32 | 1.27 | 1.37 | <0.001 |  |
| Anticoagulant history |  |  |  |  |  |  | 0.016 |
| No | 287302 | 87.5 | 1.34 | 1.30 | 1.37 | <0.001 |  |
| Yes | 40903 | 12.5 | 1.25 | 1.19 | 1.31 | <0.001 |  |

Abbreviations: OR: Odds Ratio.

**Supplementary Table 9. Sensitivity analysis between IR-related indices and the risk of pan-cancer incidence and pan-cancer-specific mortality with participants exclude pan-cancer occurred within 1 years.**

| **Characteristic** | **pan-cancer incidence** | | **pan-cancer-specific mortality** | |
| --- | --- | --- | --- | --- |
|  | HR (95% CI) | *P*-value | HR (95% CI) | *P*-value |
| **TyG Index** |  |  |  |  |
| Q1 (0-25%) | Reference |  | Reference |  |
| Q2 (25-50%) | 1.14 (1.11, 1.17) | < 0.001 | 1.23 (1.16, 1.30) | < 0.001 |
| Q3 (50-75%) | 1.25 (1.22, 1.29) | < 0.001 | 1.45 (1.37, 1.53) | < 0.001 |
| Q4 (75-100%) | 1.28 (1.25, 1.32) | < 0.001 | 1.64 (1.55, 1.73) | < 0.001 |
| **TyG-BMI Index** |  |  |  |  |
| Q1 (0-25%) | Reference |  | Reference |  |
| Q2 (25-50%) | 1.16 (1.13, 1.19) | < 0.001 | 1.22 (1.15, 1.30) | < 0.001 |
| Q3 (50-75%) | 1.24 (1.21, 1.27) | < 0.001 | 1.42 (1.34, 1.51) | < 0.001 |
| Q4 (75-100%) | 1.25 (1.22, 1.28) | < 0.001 | 1.63 (1.55, 1.73) | < 0.001 |
| **TyG-WC Index** |  |  |  |  |
| Q1 (0-25%) | Reference |  | Reference |  |
| Q2 (25-50%) | 1.24 (1.21, 1.28) | < 0.001 | 1.36 (1.28, 1.44) | < 0.001 |
| Q3 (50-75%) | 1.39 (1.36, 1.43) | < 0.001 | 1.55 (1.46, 1.64) | < 0.001 |
| Q4 (75-100%) | 1.52 (1.48, 1.56) | < 0.001 | 2.10 (1.98, 2.22) | < 0.001 |
| **TyG-WHtR Index** |  |  |  |  |
| Q1 (0-25%) | Reference |  | Reference |  |
| Q2 (25-50%) | 1.23 (1.19, 1.26) | < 0.001 | 1.29 (1.22, 1.38) | < 0.001 |
| Q3 (50-75%) | 1.35 (1.32, 1.39) | < 0.001 | 1.57 (1.48, 1.67) | < 0.001 |
| Q4 (75-100%) | 1.46 (1.42, 1.50) | < 0.001 | 2.03 (1.91, 2.15) | < 0.001 |

Model was the raw models without adjustment.

Abbreviations: HR:hazard ratio;CI :confidence interval;TyG:triglyceride-glucose index;BMI:body mass index; WC:waist circumference; WHtR:weight-to-height ratio.

**Supplementary Table 10. Sensitivity analysis between IR-related indices and the risk of pan-cancer incidence and pan-cancer-specific mortality with participants exclude pan-cancer occurred within 2 years.**

| **Characteristic** | **pan-cancer incidence** | | **pan-cancer-specific mortality** | |
| --- | --- | --- | --- | --- |
|  | HR (95% CI) | *P*-value | HR (95% CI) | *P*-value |
| **TyG Index** |  |  |  |  |
| Q1 (0-25%) | Reference |  | Reference |  |
| Q2 (25-50%) | 1.13 (1.10, 1.17) | < 0.001 | 1.23 (1.16, 1.31) | < 0.001 |
| Q3 (50-75%) | 1.25 (1.22, 1.29) | < 0.001 | 1.45 (1.35, 1.53) | < 0.001 |
| Q4 (75-100%) | 1.28 (1.25, 1.32) | < 0.001 | 1.63 (1.54, 1.73) | < 0.001 |
| **TyG-BMI Index** |  |  |  |  |
| Q1 (0-25%) | Reference |  | Reference |  |
| Q2 (25-50%) | 1.16 (1.13, 1.19) | < 0.001 | 1.23 (1.16, 1.31) | < 0.001 |
| Q3 (50-75%) | 1.24 (1.21, 1.28) | < 0.001 | 1.42 (1.34, 1.51) | < 0.001 |
| Q4 (75-100%) | 1.26 (1.22, 1.29) | < 0.001 | 1.64 (1.54, 1.74) | < 0.001 |
| **TyG-WC Index** |  |  |  |  |
| Q1 (0-25%) | Reference |  | Reference |  |
| Q2 (25-50%) | 1.24 (1.21, 1.28) | < 0.001 | 1.35 (1.27, 1.44) | < 0.001 |
| Q3 (50-75%) | 1.40 (1.36, 1.44) | < 0.001 | 1.56 (1.47, 1.66) | < 0.001 |
| Q4 (75-100%) | 1.53 (1.49, 1.57) | < 0.001 | 2.09 (1.97, 2.22) | < 0.001 |
| **TyG-WHtR Index** |  |  |  |  |
| Q1 (0-25%) | Reference |  | Reference |  |
| Q2 (25-50%) | 1.23 (1.19, 1.26) | < 0.001 | 1.29 (1.21, 1.38) | < 0.001 |
| Q3 (50-75%) | 1.36 (1.32, 1.39) | < 0.001 | 1.57 (1.48, 1.67) | < 0.001 |
| Q4 (75-100%) | 1.46 (1.42, 1.50) | < 0.001 | 2.03 (1.91, 2.16) | < 0.001 |

Model was the raw models without adjustment.

Abbreviations: HR:hazard ratio;CI :confidence interval;TyG:triglyceride-glucose index;BMI:body mass index; WC:waist circumference; WHtR:weight-to-height ratio.

**Supplementary Table11. Sensitivity analysis between IR-related indices and the risk of pan-cancer incidence and pan-cancer-specific mortality with participants exclude pan-cancer occurred within 1 years.**

| **Characteristic** | **pan-cancer incidence** | | **pan-cancer-specific mortality** | |
| --- | --- | --- | --- | --- |
|  | HR (95% CI) | *P*-value | HR (95% CI) | *P*-value |
| **TyG Index** |  |  |  |  |
| Q1 (0-25%) | Reference |  | Reference |  |
| Q2 (25-50%) | 0.99 (0.97,1.02) | 0.552 | 1.02 (0.96, 1.08) | 0.531 |
| Q3 (50-75%) | 1.02 (1.00,1.05) | 0.070 | 1.09 (1.03, 1.16) | 0.003 |
| Q4 (75-100%) | 1.01 (0.99,1.04) | 0.354 | 1.14 (1.08, 1.21) | < 0.001 |
| **TyG-BMI Index** |  |  |  |  |
| Q1 (0-25%) | Reference |  | Reference |  |
| Q2 (25-50%) | 1.00 (0.98,1.03) | 0.897 | 1.02 (0.96, 1.08) | 0.543 |
| Q3 (50-75%) | 1.03 (1.00,1.05) | 0.051 | 1.10 (1.03, 1.16) | 0.002 |
| Q4 (75-100%) | 1.05 (1.02,1.08) | < 0.001 | 1.24 (1.16, 1.31) | < 0.001 |
| **TyG-WC Index** |  |  |  |  |
| Q1 (0-25%) | Reference |  | Reference |  |
| Q2 (25-50%) | 1.06 (1.03, 1.09) | < 0.001 | 1.11 (1.04, 1.18) | 0.002 |
| Q3 (50-75%) | 1.09 (1.06, 1.12) | < 0.001 | 1.13 (1.06, 1.21) | < 0.001 |
| Q4 (75-100%) | 1.15 (1.11, 1.18) | < 0.001 | 1,41 (1.32, 1.51) | < 0.001 |
| **TyG-WHtR Index** |  |  |  |  |
| Q1 (0-25%) | Reference |  | Reference |  |
| Q2 (25-50%) | 1.03 (1.01, 1.06) | 0.017 | 1.04 (0.97, 1.10) | 0.276 |
| Q3 (50-75%) | 1.05 (1.03, 1.08) | < 0.001 | 1.12 (1.05, 1.19) | < 0.001 |
| Q4 (75-100%) | 1.10 (1.07, 1.13) | < 0.001 | 1.32 (1.24, 1.41) | < 0.001 |

Model was fully adjusted for age, sex, Townsend index, qualification, ethnicity,recruit center, alcohol consumption, smoking status, diabetes history, hypertension history ,anticoagulant history, family history of cancer, cardiovascular disease, stroke, and heart failure history.

Abbreviations: HR:hazard ratio;CI :confidence interval;TyG:triglyceride-glucose index;BMI:body mass index; WC:waist circumference; WHtR:weight-to-height ratio.

**Supplementary Table 12. Sensitivity analysis between IR-related indices and the risk of pan-cancer incidence and pan-cancer-specific mortality with participants exclude pan-cancer occurred within 2 years.**

| **Characteristic** | **pan-cancer incidence** | | **pan-cancer-specific mortality** | |
| --- | --- | --- | --- | --- |
|  | HR (95% CI) | *P*-value | HR (95% CI) | *P*-value |
| **TyG Index** |  |  |  |  |
| Q1 (0-25%) | Reference |  | Reference |  |
| Q2 (25-50%) | 0.99 (0.96, 1.02) | 0.396 | 1.02 (0.96, 1.08) | 0.582 |
| Q3 (50-75%) | 1.02 (1.00, 1.05) | 0.096 | 1.08 (1.02, 1.15) | 0.011 |
| Q4 (75-100%) | 1.01 (0.98, 1.04) | 0.432 | 1.13 (1.07, 1.21) | < 0.001 |
| **TyG-BMI Index** |  |  |  |  |
| Q1 (0-25%) | Reference |  | Reference |  |
| Q2 (25-50%) | 1.00 (0.98, 1.03) | 0.794 | 1.02 (0.96, 1.09) | 0.501 |
| Q3 (50-75%) | 1.03 (1.00, 1.06) | 0.047 | 1.09 (1.03, 1.16) | 0.005 |
| Q4 (75-100%) | 1.06 (1.03, 1.09) | < 0.001 | 1.23 (1.16, 1.31) | < 0.001 |
| **TyG-WC Index** |  |  |  |  |
| Q1 (0-25%) | Reference |  | Reference |  |
| Q2 (25-50%) | 1.05 (1.02, 1.08) | < 0.001 | 1.10 (1.03, 1.18) | 0.004 |
| Q3 (50-75%) | 1.10 (1.06, 1.13) | < 0.001 | 1.14 (1.07, 1.22) | < 0.001 |
| Q4 (75-100%) | 1.15 (1.11, 1.19) | < 0.001 | 1,40 (1.31, 1.50) | < 0.001 |
| **TyG-WHtR Index** |  |  |  |  |
| Q1 (0-25%) | Reference |  | Reference |  |
| Q2 (25-50%) | 1.03 (1.01, 1.06) | 0.020 | 1.03 (0.97, 1.11) | 0.317 |
| Q3 (50-75%) | 1.06 (1.03, 1.09) | < 0.001 | 1.12 (1.05, 1.19) | < 0.001 |
| Q4 (75-100%) | 1.10 (1.07, 1.14) | < 0.001 | 1.32 (1.24, 1.41) | < 0.001 |

Model was fully adjusted for age, sex, Townsend index, qualification, ethnicity,recruit center, alcohol consumption, smoking status, diabetes history, hypertension history ,anticoagulant history, family history of cancer, cardiovascular disease, stroke, and heart failure history.

Abbreviations: HR:hazard ratio;CI :confidence interval;TyG:triglyceride-glucose index;BMI:body mass index; WC:waist circumference; WHtR:weight-to-height ratio.
